# Adult weight change and premenopausal breast cancer risk: A prospective pooled analysis of data from 628,463 women

**DOI:** 10.1002/ijc.32892

**Published:** 2020-02-15

**Authors:** Minouk J. Schoemaker, Hazel B. Nichols, Lauren B. Wright, Mark N. Brook, Michael E. Jones, Katie M. O'Brien, Hans‐Olov Adami, Laura Baglietto, Leslie Bernstein, Kimberly A. Bertrand, Marie‐Christine Boutron‐Ruault, Yu Chen, Avonne E. Connor, Laure Dossus, A. Heather Eliassen, Graham G. Giles, Inger T. Gram, Susan E. Hankinson, Rudolf Kaaks, Timothy J. Key, Victoria A. Kirsh, Cari M. Kitahara, Susanna C. Larsson, Martha Linet, Huiyan Ma, Roger L. Milne, Kotaro Ozasa, Julie R. Palmer, Elio Riboli, Thomas E. Rohan, Carlotta Sacerdote, Atsuko Sadakane, Malin Sund, Rulla M. Tamimi, Antonia Trichopoulou, Giske Ursin, Kala Visvanathan, Elisabete Weiderpass, Walter C. Willett, Alicja Wolk, Anne Zeleniuch‐Jacquotte, Dale P. Sandler, Anthony J. Swerdlow

**Affiliations:** ^1^ Division of Genetics and Epidemiology The Institute of Cancer Research London United Kingdom; ^2^ Department of Epidemiology University of North Carolina Gillings School of Global Public Health Chapel Hill NC; ^3^ Biostatistics and Computational Biology Branch National Institute of Environmental Health Sciences, National Institutes of Health Durham NC; ^4^ Department of Medical Epidemiology and Biostatistics (MEB) Karolinska Institutet Stockholm Sweden; ^5^ Clinical Effectiveness Research Group Institute of Health and Society, University of Oslo Oslo Norway; ^6^ Department of Clinical and Experimental Medicine University of Pisa Pisa Italy; ^7^ Department of Population Sciences Beckman Research Institute of City of Hope Duarte CA; ^8^ Slone Epidemiology Center at Boston University Boston MA; ^9^ Department of Population Health and Perlmutter Cancer Center New York University School of Medicine New York NY; ^10^ Department of Epidemiology Johns Hopkins Bloomberg School of Public Health Baltimore MD; ^11^ Johns Hopkins Sidney Kimmel Comprehensive Cancer Center Baltimore MD; ^12^ Nutrition and Metabolism Section International Agency for Research on Cancer Lyon France; ^13^ Department of Epidemiology Harvard T.H. Chan School of Public Health Boston MA; ^14^ Channing Division of Network Medicine, Department of Medicine Brigham and Women's Hospital and Harvard Medical School Boston MA; ^15^ Cancer Epidemiology and Intelligence Division Cancer Council Victoria Melbourne VIC Australia; ^16^ Centre for Epidemiology and Biostatistics School of Population and Global Health, The University of Melbourne Melbourne VIC Australia; ^17^ Department of Community Medicine, Faculty of Health Sciences University of Tromsø (UiT), The Arctic University of Norway Tromsø Norway; ^18^ Department of Biostatistics and Epidemiology School of Public Health and Health Sciences, University of Massachusetts Amherst MA; ^19^ Division of Cancer Epidemiology, DKFZ Heidelberg Germany; ^20^ Nuffield Department of Population Health University of Oxford Oxford United Kingdom; ^21^ Ontario Institute for Cancer Research Toronto ON Canada; ^22^ Radiation Epidemiology Branch, Division of Cancer Epidemiology and Genetics National Cancer Institute Bethesda MD; ^23^ Karolinska Institute, Institute of Environmental Medicine Stockholm Sweden; ^24^ Radiation Effects Research Foundation Hiroshima Japan; ^25^ School of Public Health Imperial College London United Kingdom; ^26^ Albert Einstein College of Medicine Bronx NY; ^27^ Unit of Cancer Epidemiology Città della Salute e della Scienza University‐Hospital and Center for Cancer Prevention (CPO) Turin Italy; ^28^ Department of Surgical and Perioperative Sciences Umeå University Umeå Sweden; ^29^ Hellenic Health Foundation Athens Greece; ^30^ Cancer Registry of Norway, Institute of Population‐Based Cancer Research Oslo Norway; ^31^ Institute of Basic Medical Sciences, University of Oslo Oslo Norway; ^32^ Department of Preventive Medicine University of Southern California Los Angeles CA; ^33^ Johns Hopkins School of Medicine Baltimore MD; ^34^ International Agency for Research on Cancer (IARC)/World Health Organization (WHO) Lyon France; ^35^ Department of Surgical Sciences Uppsala University Uppsala Sweden; ^36^ Epidemiology Branch National Institute of Environmental Health Sciences, National Institutes of Health Durham NC; ^37^ Division of Breast Cancer Research The Institute of Cancer Research London United Kingdom

**Keywords:** breast neoplasms, premenopause, body weight changes, risk factors, cohort studies

## Abstract

Early‐adulthood body size is strongly inversely associated with risk of premenopausal breast cancer. It is unclear whether subsequent changes in weight affect risk. We pooled individual‐level data from 17 prospective studies to investigate the association of weight change with premenopausal breast cancer risk, considering strata of initial weight, timing of weight change, other breast cancer risk factors and breast cancer subtype. Hazard ratios (HR) and 95% confidence intervals (CI) were obtained using Cox regression. Among 628,463 women, 10,886 were diagnosed with breast cancer before menopause. Models adjusted for initial weight at ages 18–24 years and other breast cancer risk factors showed that weight gain from ages 18–24 to 35–44 or to 45–54 years was inversely associated with breast cancer overall (e.g., HR per 5 kg to ages 45–54: 0.96, 95% CI: 0.95–0.98) and with oestrogen‐receptor(ER)‐positive breast cancer (HR per 5 kg to ages 45–54: 0.96, 95% CI: 0.94–0.98). Weight gain from ages 25–34 was inversely associated with ER‐positive breast cancer only and weight gain from ages 35–44 was not associated with risk. None of these weight gains were associated with ER‐negative breast cancer. Weight *loss* was not consistently associated with overall or ER‐specific risk after adjusting for initial weight. Weight increase from early‐adulthood to ages 45–54 years is associated with a reduced premenopausal breast cancer risk independently of early‐adulthood weight. Biological explanations are needed to account for these two separate factors.

## Abstract 2

**What's new?**

Body weight in childhood and early adulthood plays a key role in determining premenopausal breast cancer risk but little is conclusively known about how subsequent weight changes affect this risk. Here the authors pooled results from existing studies on weight changes and breast cancer risk including more than 600,000 premenopausal women. The results show that weight gain >10–15 kg from early adulthood on lowers the risk of developing premenopausal breast cancer, providing further evidence of body weight as an important determinant of breast cancer risk.

## Introduction

The influence of obesity on breast cancer risk varies by life‐stage. Adiposity before menopause is inversely associated with risk, whereas adiposity during the postmenopausal years is positively associated with risk.^1^, ^2^, ^3^ The inverse association with premenopausal adiposity is particularly strong for adiposity in early adulthood, that is, ages 18–24 years,^4^ and it is likely that the origin of this association lies in childhood.^5^, ^6^

It is not clear whether changes in weight after early adulthood further affect risk of premenopausal breast cancer. The role of weight gain in adulthood is of interest because increases in body weight during adulthood mostly reflect accumulation of adipose rather than lean tissue, and therefore any change might reflect body fatness better than adult weight itself, and because of its association with intra‐abdominal fat deposition, which is more metabolically active than peripheral adiposity.^7^ Timing of weight change might additionally be relevant in that weight change at different stages of life, for example, during periods of hormonal change such as during pregnancy, might have different biological effects and differentially affect breast cancer risk.^8^

Due to the relatively low incidence of premenopausal breast cancer, past studies have had limited numbers of cases to investigate the association of weight change with risk by timing of weight change, to examine the effect of weight loss or to analyse these associations by participant or tumour characteristics. Additionally, past studies have often only used proxies for menopausal status, such as status at study entry or attained age, rather than time‐updated menopausal status.

We pooled individual‐level data from prospective studies to investigate the association of weight change and its timing with premenopausal breast cancer risk, overall and by breast cancer characteristics.

## Materials and Methods

We used data from 17 of the 22 cohort studies in the Premenopausal Breast Cancer Collaborative Group^9^ that had participants' weight available at a minimum of two time points before women were age 55 years and had at least 100 breast cancer cases diagnosed before age 55 years. Individual‐level data were pooled from cohorts in North America (*n* = 9), Europe (*n* = 6), Asia (*n* = 1) and Australia (*n* = 1), with participants recruited between 1963 and 2013. Data from 1 to 16 questionnaire rounds per study were harmonised to a common protocol. Women provided historical information on their weight prior to study entry on the baseline questionnaire and their current body weight was provided or measured at baseline and on follow‐up questionnaires (if available). This work was approved by the relevant institutional review boards and women provided informed consent to partake.

Women were included in the analysis if they were breast cancer‐free and premenopausal at study entry, and had premenopausal weight available for at least two age categories (defined below). Premenopausal follow‐up time was determined from menopause information obtained from multiple questionnaire rounds, and, if missing, assumptions based on attained age using age 50 as cut‐off (Supporting Information).

The main analytical endpoint was diagnosis with invasive or *in situ* premenopausal breast cancer combined. We also conducted analyses of invasive and *in situ* outcomes separately, as well as outcomes by hormone‐receptor status and a clinicopathological surrogate definition of intrinsic breast cancer subtypes (Supporting Information).

Analyses were conducted using STATA 14.2.^10^ Data on weight was available at 2–13 time points per study. We first investigated weight patterns across time with longitudinal trajectory models at a selected number of time points^11^ (Supporting Information Figs. S1 and S2). These models resulted in trajectories of weight gain delineated by initial weight, but did not delineate women with weight loss as a separate group. We therefore instead constructed variables for weight change between the age categories 18–24, 25–34, 35–44 and 45–54 years, to be able to use data from all the studies with varying numbers of time points and to examine the association of weight loss with risk. Weight at ages 18–24 was derived, for the great majority of subjects, from weight at age 18 or 20 (depending on the study) retrospectively reported on the baseline questionnaire. Weight at other ages was usually reported or measured at or after recruitment to the study.

Follow‐up for breast cancer began at the second weight assessment that was used to compute weight change, or at recruitment, whichever was later. Follow‐up ended with the first of the following events: breast cancer diagnosis, menopause, last follow‐up, death or age 55 years. Hazard ratios (HR) and 95% confidence intervals (CI) representing estimates of relative risk of breast cancer were derived from Cox proportional hazards models with attained age as the underlying time scale.^12^ Models were adjusted for cohort, year of birth, age at menarche, parity, age at first birth, time since most recent birth, adult height at recruitment and family history of breast cancer. Covariate information was updated over follow‐up, where available. In the main analyses, we analysed weight change in categories of 5.0 kg increments and as linear trends in risk per 5.0 kg of weight gain. We also obtained results in finer strata of 2.5 kg increments (Supporting Information). We obtained HRs for weight change with and without adjustment for starting weight to investigate whether starting weight was a confounder, but presented results adjusted for starting weight unless otherwise stated.

Tests for effect modification of weight change by cohort, starting weight, other available breast cancer risk factors and time since weight change were conducted using log‐likelihood ratio tests.^13^ We estimated separate risk‐factor associations for breast cancer type‐specific outcomes using an augmentation method.^14^

We conducted sensitivity analyses by (*i*) excluding the first 2 years of follow‐up to reduce the chance of reverse causation by preclinical disease; (*ii*) restricting analyses to reported, rather than assumed, premenopausal follow‐up time; (*iii*) repeating the analyses excluding one study at a time; (*iv*) additionally adjusting for the number of years between weight assessments; (*v*) in analyses restricted to subjects with weight at ages 18–24 available, adjusting for weight at ages 18–24 rather than weight at the start of the age category; (*vi*) restricting analyses to the five cohorts contributing to analyses of weight change at all of the six age groups; (*vii*) repeating analyses for subjects with nonmissing covariate information; (*viii*) for subjects with available data, additionally adjusting one at a time for variables only available for some cohorts: ethnicity, cigarette smoking, alcohol consumption, physical activity level, polycystic ovary syndrome, childhood somatotype and mammographic screening history.

We additionally analysed the average annual weight change assuming a linear trajectory as a risk factor, rather than an absolute increase in weight.

### Data availability

Research data will be made available upon reasonable request due to privacy/ethical restrictions.

## Results

The analyses included 628,463 women, whose median age at recruitment was 39.4 (interquartile range 33.8–44.0) years and who were followed for a median of 10.1 (interquartile range 5.9–15.5) years from recruitment during which 10,886 breast cancer cases (8,509 invasive) were diagnosed. Oestrogen‐receptor (ER) status was known for 6,994 (72.5% of invasive and 43.5% of *in situ*) breast cancer patients; ER, progesterone‐receptor (PR) and HER2‐status was available for 3,425 (37.2% invasive, 13.9% *in situ*) breast cancer patients.

Most women were white (85.7%), from North America (56.6%), or Europe (41.1%) (Table 1). Women in the weight loss group were on average heavier at the onset than women who gained weight. Most women were parous at recruitment (80.7%) and 12.4% had a family history of breast cancer. The age‐specific weight change variables were available for 5–14 cohorts per variable, representing weight change over median time intervals of 6.1–28.2 years and median follow‐up for breast cancer of 4.2–17.2 years (Supporting Information Tables S1 and S2). For all follow‐up periods, the majority (80.3–90.4%) of women gained weight.

**Table 1:** Characteristics of women included in the analyses, by degree of weight change between the earliest available weight and weight at or close to recruitment to the study

Factor^1^		Weight change category^2^						Overall
		Loss ≥5 kg	Stable (±4.9 kg)	Gain 5–9.9 kg	Gain 10–14.9 kg	Gain 15–19.9 kg	Gain ≥20 kg	
Height (cm)	Mean	165.0	164.4	164.9	165.0	165.2	165.6	164.8
Age at first weight^3^	Mean	19.9	22.1	20.1	19.5	19.3	19.1	20.6
First weight (kg)	Mean	71.4	57.5	56.4	56.7	57.8	60.5	58.2
First BMI (kg/m^2^)	Mean	26.2	21.3	20.7	20.8	21.2	22.0	21.4
Age at recruitment (years)	Mean	37.9	37.6	38.8	39.8	40.2	40.4	39.3
Recruitment weight (kg)	Mean	60.6	58.5	63.6	68.8	74.8	89.2	65.7
Recruitment BMI (kg/m^2^)	Mean	22.2	21.7	23.4	25.3	27.4	32.5	24.2
Change in weight between starting age and recruitment (kg)	Mean	−10.8	1.0	7.2	12.1	17.0	28.7	7.5
	Median	−8.7	1.4	7.0	12.0	17.0	26.0	5.9
Rate of weight change between starting age and recruitment (kg/year)	Mean	−0.8	0.1	0.5	0.7	0.9	1.5	0.4
	Median	−0.5	0.1	0.4	0.6	0.8	1.3	0.3
Ethnicity								
White	%	90.0	90.4	87.7	84.4	79.6	70.5	85.7
Black	%	6.0	5.3	8.6	12.3	17.5	27.3	10.6
Asian	%	1.9	2.8	2.1	1.8	1.2	0.5	2.0
Other	%	2.1	1.6	1.6	1.6	1.7	1.7	1.6
Continent of residence								
North America	%	63.2	57.4	53.2	51.6	55.0	65.6	56.6
Europe	%	35.3	40.8	44.9	46.1	42.6	32.1	41.4
Australia	%	0.8	0.7	1.3	1.8	2.2	2.2	1.3
Asia	%	0.7	1.1	0.7	0.5	0.3	0.09	0.7
All participants	Total	32,726	253,164	140,227	86,632	48,297	67,417	628,463

1 Frequency distributions for nonmissing values only.

2 Weight change was computed between earliest available weight and first weight available at or after recruitment, with the exception of a small number of subjects for whom weight change was computed from two retrospectively assessed weights before recruitment because weight at or after recruitment was not available.

3 Weight was retrospectively assessed at age 18 or 20 for the majority of studies.

**Figure 1:**
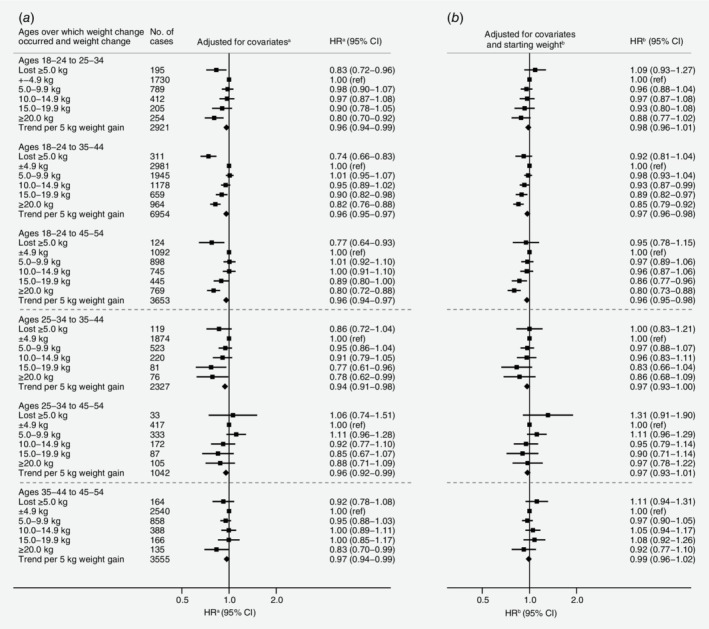
Relative risk of premenopausal breast cancer in relation to weight change between various ages. Abbreviations: HR, hazard ratio; CI, confidence interval. (*a*) Adjusted for attained age, cohort, year of birth, adult height, age at menarche, age at first birth, number of births, time since last birth and family history of breast cancer. (*b*) Adjusted for covariates in (*a*) plus weight at start of age range.

Figure 1 shows HRs in relation to weight change without (left) and with (right) adjustment for starting weight. There was an inverse U‐shaped association with risk (Fig. 1 and Supporting Information Fig. S3, left), with women who lost weight and those who gained weight since age 18–24 having lower HRs than women whose weight remained constant (within ±5.0 kg). Women who *lost* ≥5 kg since ages 18–24 years had a statistically significantly lower breast cancer risk than those whose weight remained constant. However, after additionally adjusting for starting weight (Figure 1 and Supporting Information Fig. S3, right), which was on average greater for women with weight loss than for those who gained weight, the inverse HRs for weight loss were attenuated and no longer associated with risk.

Weight *gain* from ages 18–24 to 35–44 years or from ages 18–24 to 45–54 years was associated with lower breast cancer risk, although HRs were somewhat attenuated with adjustment for starting weight. Linear inverse trends in risk per 5 kg gain over these time periods remained statistically significant (HRs: 0.97, 95% CI: 0.96–0.98 for ages 18–24 to 35–44 and 0.96, 95% CI: 0.95–0.98 for 18–24 to 45–54 years). The association of starting weight with risk remained statistically significant in these models (data not shown).

Patterns of risk with weight change from later ages (i.e. from 25 to 34 or 35–44 years) were less clear, with no association between weight loss and linear trends with weight gain not being statistically significantly associated with risk after adjustment for starting weight.

In analyses by breast cancer invasiveness, the inverse associations of weight gain with breast cancer risk tended to be stronger for *in situ* than for invasive breast cancer, but only significantly so for weight gain between ages 18–24 and 25–34 years (*p*‐interaction = 0.007) (Supporting Information Table S3). Stronger associations for *in situ* than invasive breast cancer were also observed when we repeated the analyses among subjects with a previous history of mammographic screening only (data not shown).

Associations of weight gain from young ages tended to be stronger for ER‐positive than ER‐negative (Table 2) or for ER+/PR+ than ER− and PR− breast cancer (Supporting Information Table S4). The differences in hazard ratios between these subgroups were only strongly statistically significant for one weight change group, however.

**Table 2:** Risk of premenopausal breast cancer in relation to weight change between various ages, by oestrogen‐receptor status of breast cancer

Ages at weight change	Median weight change, kg (25–75th percentile)	ER status	No. of cases	Weight gain category, kg							Trend per 5 kg gain^1^	
				Loss ≥5.0 kg	Stable ±4.9 kg	Gain 5.0–9.9 kg	Gain 10.0–14.9 kg	Gain 15.0–19.9 kg	Gain ≥20.0 kg			
				HR (95% CI)^2^	HR^2^	HR (95% CI)^2^	HR (95% CI)^2^	HR (95% CI)^2^	HR (95% CI)^2^	*p*‐int	HR (95% CI)^2^	*p* int‐trend
Ages 18–24 to 25–34	4.5 (0.4–9.6)	ER+	1834	1.00 (0.80–1.25)	1.00 (ref)	0.89 (0.79–1.01)	0.96 (0.83–1.12)	0.87 (0.71–1.07)	0.85 (0.70–1.02)		0.98 (0.95–1.01)	
		ER−	591	1.24 (0.86–1.79)	1.00 (ref)	1.10 (0.90–1.36)	1.06 (0.82–1.38)	0.85 (0.59–1.24)	1.12 (0.83–1.50)	0.39	1.01 (0.96–1.07)	0.24
Ages 18–24 to 35–44	6.9 (2.3–13.6)	ER+	3,976	0.92 (0.78–1.09)	1.00 (ref)	1.00 (0.92–1.08)	0.96 (0.87–1.05)	0.88 (0.78–0.99)	0.77 (0.69–0.86)		0.95 (0.93–0.97)	
		ER−	1,268	0.85 (0.62–1.18)	1.00 (ref)	1.07 (0.93–1.24)	1.11 (0.94–1.31)	1.11 (0.90–1.35)	1.01 (0.85–1.21)	0.07	1.01 (0.98–1.04)	0.0009
Ages 18–24 to 45–54	10.0 (4.5–18.2)	ER+	2,249	0.80 (0.60–1.05)	1.00 (ref)	0.98 (0.87–1.11)	1.02 (0.90–1.15)	0.89 (0.77–1.03)	0.79 (0.70–0.90)		0.96 (0.94–0.98)	
		ER−	625	1.27 (0.81–2.00)	1.00 (ref)	1.26 (1.00–1.58)	1.18 (0.93–1.52)	0.97 (0.73–1.31)	1.02 (0.80–1.30)	0.27	0.99 (0.95–1.02)	0.16
Age 25–34 to 35–44	3.2 (0–7.3)	ER+	1,547	0.99 (0.77–1.28)	1.00 (ref)	0.99 (0.86–1.13)	0.99 (0.81–1.20)	0.71 (0.50–0.99)	0.72 (0.50–1.03)		0.94 (0.89–0.99)	
		ER−	466	0.74 (0.44–1.24)	1.00 (ref)	1.04 (0.82–1.33)	1.13 (0.81–1.57)	0.85 (0.49–1.49)	1.08 (0.64–1.83)	0.61	1.03 (0.96–1.10)	0.03
Age 25–34 to 45–54	7.3 (3.1–13.6)	ER+	726	1.45 (0.94–2.26)	1.00 (ref)	1.10 (0.92–1.32)	0.91 (0.73–1.15)	0.86 (0.64–1.16)	0.90 (0.68–1.19)		0.95 (0.90–1.00)	
		ER−	169	0.25 (0.03–1.80)	1.00 (ref)	1.07 (0.74–1.54)	0.91 (0.58–1.43)	0.98 (0.56–1.71)	0.69 (0.37–1.28)	0.58	0.95 (0.86–1.04)	1.00
Age 35–44 to 45–54	3.2 (0–7.7)	ER+	2,575	1.15 (0.94–1.41)	1.00 (ref)	0.95 (0.86–1.05)	1.08 (0.94–1.24)	1.16 (0.96–1.42)	0.89 (0.71–1.12)		1.00 (0.96–1.03)	
		ER−	670	0.77 (0.48–1.22)	1.00 (ref)	1.04 (0.86–1.26)	1.03 (0.79–1.35)	0.71 (0.44–1.14)	0.89 (0.58–1.37)	0.19	0.98 (0.91–1.05)	0.61

1 Including subjects with weight gain only.

2 HRs are adjusted for attained age, cohort, year of birth, adult height, weight at start of age range, age at menarche, age at first birth, number of births, time since last birth and family history of breast cancer.

Abbreviations: CI, confidence interval; ER, oestrogen‐receptor; HR, hazard ratio; *p*‐int, *p*‐value for interaction test.

In analysis by breast cancer intrinsic subtype, weight gain from ages 18–24 onwards was inversely associated with Luminal A‐like (ER+ PR+ HER2−) breast cancer and weight gain from ages 18–24 to 35–44 and to 45–54 years additionally with luminal B‐like (other ER+/PR+) breast cancer risk (Supporting Information Fig. S4). For some of the age groups, there was evidence of a positive association with nonluminal (ER− PR−) breast cancer risk, in particular, HER2‐enriched breast cancer, whereas there was no association with triple‐negative breast cancer risk.

There was evidence for effect modification by starting weight for the linear effect of weight change at two age groups, ages 18–24 to 45–54 years (*p*‐interaction = 0.02) and 25–34 to 35–44 years (*p*‐interaction = 0.006), but in opposite directions and some of the results were based on small numbers of cases (Supporting Information Table S5). We observed no statistically significant evidence for effect modification in the linear association of weight change with risk by other breast cancer risk factors considered (childhood body shape, adult height, age at menarche, parity, age at first birth, number of births, family history of breast cancer and ethnicity, see Supporting Information Table S6) or by time since weight change (Supporting Information Table S7).

The main findings did not materially differ in the sensitivity analyses conducted (see methods and Supporting Information Tables S8 and S9 for selected results) except that the inverse associations with weight gain were somewhat stronger when a stricter definition of menopausal status was applied. When analysing risk in relation to average annual weight change, rather than absolute amount of weight change, conclusions were similar, with the strongest inverse association with risk observed for weight change over the longest interval, from ages 18–24 to 45–54 years (HR = 0.82, 95% CI: 0.75–0.89 per kg/year), reflecting the largest absolute weight gains (Supporting Information Fig. S5).

## Discussion

We observed that both weight loss ≥5 kg and weight gain of ≥10–15 kg since early adulthood were inversely associated with premenopausal breast cancer risk, but that degree of weight change was associated with initial weight, and that only weight *gain* was associated with risk after controlling for early adult weight. Early‐adulthood weight remained significant in such models, indicating that both starting weight and weight gain are associated with risk. There was weak and inconsistent evidence that the effect of weight gain depended on starting weight, and no evidence that the association varied by other investigated breast cancer risk factors. We captured weight change between ages 18–24 and 35–44 years, when most parous women had their pregnancies, but did not find statistical evidence that the association of weight change with risk differed between parous and nulliparous women. Weight change from later ages, age 35 years onwards, was not associated with risk.

Our pooling project incorporates data from most,^15^, ^16^, ^17^, ^18^, ^19^, ^20^, ^21^, ^22^ although not all,^23^, ^24^, ^25^, ^26^, ^27^, ^28^ published prospective studies on long‐term weight change and premenopausal breast cancer risk, and additionally include previously unpublished data. It consequently had enhanced statistical power based on its large sample size. Few past studies reported on weight loss separately; those that did reported null associations or nonsignificant inverse associations with weight loss since age 18 or 20 years compared with women whose weight remained stable,^15^, ^21^, ^22^, ^23^, ^26^ and not all adjusted for starting weight. In relation to weight gain, the majority of prospective studies have reported null or nonstatistically significant inverse linear trends,^15^, ^18^, ^19^, ^20^, ^21^, ^22^, ^23^, ^24^, ^25^, ^26^, ^27^, ^28^ except for two reporting positive associations, but with no clear dose–response relationship.^16^, ^20^

There was a tendency for inverse associations with risk to be somewhat stronger for *in situ* than invasive breast cancer; this might reflect stage‐specific aetiology or could be artefactual, for example, a deficit of *in situ* diagnoses could occur if increasing weight made women less likely to attend breast screening or if they presented later because breast self‐examination and lump detection is more difficult.^29^ Stronger associations for *in situ* than invasive cancer were also observed among women who had previously had a screening mammogram, suggesting that it is not explained by past breast screening attendance, but unfortunately we did not have data on mode of detection of breast cancer.

In analyses by ER‐status, we observed inverse associations of weight gain with ER+, but not with PR–breast cancer. This agrees with our previous finding that BMI at ages over 25 years is inversely associated with risk of hormone‐receptor‐positive breast cancer only.^4^ In augmentation analyses by intrinsic subtype, however, we observed somewhat contradictory findings, with some weight change variables being positively associated with HER2‐enriched breast cancer and nonluminal breast cancer overall. These analyses were conducted on somewhat different subsets of the data and some of them on small numbers. Whether there is an association of weight change with nonluminal subtypes remains therefore uncertain.

It is of interest that we observed the strongest inverse associations with risk for weight change from early adulthood and no significant association of risk with absolute or rate of weight gain from ages 35–44 years onwards. Weight gain soon after age 18, if not followed by later weight loss, would lead to the greatest cumulative exposure to adiposity. It is possible that it is cumulative exposure to excess weight that is inversely associated with risk or that late weight gain is outside the susceptibility window for premenopausal breast cancer, for example, because there is a lag time between weight gain and an effect on risk. Our analyses by time since weight gain did not suggest the latter is the case. The lack of association with later (i.e. ≥35 years) weight gain appears discordant with the results from two previous studies. The EPIC‐PANACEA study reported a positive association of rate of weight gain over 4 years with breast cancer diagnosed at age ≤50 years, based on 283 cases.^30^ Women were premenopausal at study entry (median age 40.7 years, M. Emaus, personal communication) but no information on menopausal status at the second weight assessment or at diagnosis was available; it is therefore possible that some of the women were postmenopausal at diagnosis. The Nurses' Health Study reported a positive association of weight gain over 4 years among initially premenopausal women with breast cancer risk over the subsequent 2 years (HR: 1.38, 95% CI: 1.13–1.69 for ≥15 *vs*. <5 lbs, *n* = 736 cases).^31^ In our study, weight change was assessed over longer periods but the reason for the disparity in results is unclear.

The strong inverse association of breast cancer risk with early adult body size^4^ may originate in early life, or in childhood/adolescence.^5^, ^6^ It has been hypothesised to be due to greater differentiation of breast tissue during puberty,^2^, ^32^ altered oestrogen metabolism,^33^ lower adult mammographic density^34^, ^35^ and/or lower circulating IGF‐I levels^36^ in heavier girls. Additional weight gain is associated with a reduction in mammographic density^37^ and substantial weight gain leading to obesity suppresses ovarian function,^38^, ^39^ with a consequent reduction in endogenous sex hormone, in particular, progesterone, exposure.^38^ Weight gain might affect risk through changes in hormone profile because young women with high BMI have been reported to have lower levels of sex‐hormone‐binding globulin (SHBG), oestradiol and progesterone, and higher levels of free testosterone than women with lower BMI.^40^ Oestrogens and testosterone have been associated with premenopausal breast cancer risk,^41^ although less clearly than for postmenopausal breast cancer, but the evidence for an association of risk with progesterone is inconsistent, however.^41^ A recent study reported lower breast cell proliferation in heavier compared with leaner premenopausal women, and the reverse in postmenopausal women, which might be hormone‐related.^42^

Strengths of our study include its prospective design, its large number of cases, and therefore its ability to investigate associations according to breast cancer characteristics, multiple time‐points of weight assessments, and the use of time‐updated covariates. Limitations include that weight at ages 18–24 years was ascertained by recall for most participants, but recalled weight at age 18 years has been shown to correlate well with measured weight,^43^ and that we did not consider central adiposity measures. We studied weight change over six, some overlapping, age categories, using data of somewhat different populations, but a sensitivity analyses restricting to the five cohorts that contributed to all age categories showed similar results. There were too few women contributing to consecutive nonoverlapping time periods of weight change to investigate the role timing of weight change in a single model. In analyses by breast cancer subtype, numbers of subtype‐specific breast cancers were modest for some of the weight change variables. Furthermore, our data set was not well‐suited to investigate Asian women. We did not observe effect modification by ethnicity, but the study included relatively few women of Asian descent. It has been suggested that among Asian women, there is a positive association between BMI and premenopausal breast cancer risk,^44^ but prospective studies of weight *gain* in Asian women have, so far, shown an inverse or null association with premenopausal breast cancer risk overall.^24^, ^26^, ^45^

Our results may contribute to the understanding of breast cancer causation and aid in risk stratification. However, weight gain would not provide a strategy for long‐term risk reduction because weight and weight gain are positively associated with risks of postmenopausal breast cancer, several other types of cancer, and other adverse health outcomes.^46^, ^47^ Additionally, obese women diagnosed with breast cancer tend to have worse outcomes than leaner women, independent of their menopausal status.^48^

In conclusion, we have observed that both body size in early adulthood and subsequent weight gain are independently associated with reductions in premenopausal breast cancer risk. There is a need to understand mechanisms underlying this finding, which may provide a means for breast cancer prevention.

## Conflict of interest

Michael Jones, Minouk Schoemaker, Anthony Swerdlow and Lauren Wright declared grant funding to the Institute of Cancer Research from the charity Breast Cancer Now. Dr Kataro Ozasa declared an institutional research contract with the US National Cancer Institute. None of the other authors declared a conflict of interest with regard to this paper.

## Disclaimer

Since 1 January 2019, Elisabete Weiderpass has been a staff member of the International Agency for Research on Cancer. Where authors are identified as personnel of the International Agency for Research on Cancer/World Health Organization (E Weiderpass, L Dossus), the authors alone are responsible for the views expressed in this article and they do not necessarily represent the decisions, policy or views of the International Agency for Research on Cancer/World Health Organization.

## Supporting information


**Appendix S1**. Supporting Information.
