# Supplementary material for: Adult weight change and premenopausal breast cancer risk: A prospective pooled analysis of data from 628,463 women
Source: Int J Cancer. 2020 Feb 15;147(5):1306–14. doi: 10.1002/ijc.32892 (PMC7365745; doi:10.1002/ijc.32892)
Supplement: Supplementary file 1 — Appendix S1. Supporting Information. [file IJC-147-1306-s001.pdf]

Adult weight change and premenopausal breast cancer risk: a prospective pooled analysis in 628,463 women

Minouk J Schoemaker, Hazel B Nichols, Lauren B Wright, Mark N Brook, Michael E Jones, Katie M O'Brien, Hans-Olov Adami, Laura Baglietto, Leslie Bernstein, Kimberly A Bertrand, Marie-Christine Boutron-Ruault, Yu Chen, Avonne E Connor, Laure Dossus, A Heather Eliassen, Graham G Giles, Inger T Gram, Susan E Hankinson, Rudolf Kaaks, Timothy J Key, Victoria A Kirsh, Cari M Kitahara, Susanna C Larsson, Martha Linet, Huiyan Ma, Roger L Milne, Kotaro Ozasa, Julie R Palmer, Elio Riboli, Thomas E Rohan, Carlotta Sacerdote, Atsuko Sadakane, Malin Sund, Rulla M Tamimi, Antonia Trichopoulou, Giske Ursin, Kala Visvanathan, Elisabete Weiderpass, Walter C Willett, Alicja Wolk, Anne Zeleniuch-Jacquotte, Dale P Sandler, Anthony J Swerdlow

Table of Contents

	Page
**Supplementary Materials & Methods**	**3**
**Supplementary tables**	
Table S1: Study characteristics and numbers of premenopausal women with information on weight change, by cohort study	**5**
Table S2: Descriptive statistics on weight change between age categories in the pooled analysis	**6**
Table S3: Risk of premenopausal breast cancer in relation to weight change between various ages, by invasiveness of breast cancer	**7**
Table S4: Risk of premenopausal breast cancer in relation to weight change between various ages, by oestrogen and progesterone-receptor status of the breast cancer	**8**
Table S5: Risk of premenopausal breast cancer in relation to weight change between various ages, by initial BMI	**9**
Table S6: Risk of premenopausal breast cancer per 5 kg weight gain, by age at weight change and other breast cancer risk factors	**11**
Table S7: Risk of premenopausal breast cancer in relation to weight change category, by age at weight change and time since weight change	**13**
Table S8: Results from selected sensitivity analyses: Risk of premenopausal breast cancer in relation to weight change category, by age at weight change. For (1) all subjects included in main analysis (2) adjusting additionally for number of years between weight assessments (3) excluding first two years of follow-up (4) strictly known premenopausal time only (5) adjusting for weight at ages 18-24 in subset of women with available data.	**15**
Table S9: Risk of premenopausal breast cancer in relation to weight change between various ages, main results and results restricted to the 5 cohorts that contributed to analyses of weight change at all of the six age groups investigated	**17**

Supplementary figures

Figure S1: Trajectory modelling using 3 time points and 6 trajectories across age range 18-54 years using 15 cohorts. **18**

Figure S2: Trajectory modelling using 8 time points and 6 trajectories across age range 18-54 years using 3 cohorts. **19**

Figure S3: Relative risk of premenopausal breast cancer in relation to weight change between various ages, by 2.5kg increments **20**

Figure S4: Risk of premenopausal breast cancer in relation to weight gain per 5kg between various ages, by breast cancer intrinsic subtype **21**

Figure S5: Risk of premenopausal breast cancer in relation to the annual rate of weight change between various age groups **23**

Supplementary references **24**

Supplementary materials & methods

The collaboration

The Premenopausal Breast Cancer Collaborative Group is a collaboration of 22 prospective studies brought together to investigate the aetiology of breast cancer in premenopausal women and women under the age of 55 years. For the current paper, we used data from 17 studies that are part of the Group for which information on participants' weight was available at multiple time points. Individual-level data from baseline and follow-up questionnaire rounds were harmonised to a common template. All studies provided data for the full cohort of women recruited under the age of 55, except for the Canadian Study of Diet, Lifestyle and Health (CSDLH), which provided a case-cohort dataset. Data were available for 1-16 questionnaire rounds per study. All studies had at least two rounds except the European Prospective Investigation into Cancer and Nutrition (EPIC) study and the Canadian Study of Diet, Lifestyle and Health (CSDLH) for which only the baseline questionnaire was available. Information on weights prior to study entry recalled at the time of the baseline questionnaire and on current weight from baseline and follow-up questionnaires (where available), was used to construct the data set.

Derivation of age at menopause and premenopausal follow-up time

All cohorts provided information on menopausal status from one or more questionnaire rounds. Participants were asked whether they had had any menstrual periods during the previous 6 or 12 months, depending on study, and/or whether they believed their periods had stopped permanently. Participants were asked about the age at their last period and the reason their periods stopped. We used this information to construct premenopausal follow-up time for analysis. Age at menopause was computed for each participant based on (i) reported age at menopause or, if age was missing, (ii) age first known postmenopausal if under age 50, (iii) age last known premenopausal if over age 50 or (iv) age 50 if no information was provided. When a hysterectomy was reported as reason for the menopause, follow-up was censored at the age of hysterectomy. Since women with breast cancer often become postmenopausal due to their treatment and breast cancers diagnosed in the year of their menopause could be considered aetiologically premenopausal, the year during which women reported that they had become postmenopausal was analysed as premenopausal follow-up time. As a sensitivity analysis, we repeated the main analyses including only known premenopausal follow-up time up to the age at reported menopause (subjects under i) or, if age at menopause was missing, the age at the last questionnaire when the participant reported she was premenopausal (ii and iii).

Trajectory Modelling of weight change over time

We initially used a group-based trajectory modelling approach to investigate the pattern of weight change over time and identify subgroups within our premenopausal cohort that shared a similar underlying trajectory of body weight. This method is designed to identify relatively homogeneous clusters of developmental trajectories within the population[1]. We used the traj function provided in the statistical software package STATA[2]. The number of time points for which participants' weights were available varied between 2 and 13 per study. Due to the varying number of time points per study, we chose to model 3 time points using data from 15 cohorts and 8 time points using 3 cohorts. We fitted 6 and 10 trajectories. These models resulted in trajectories of weight gain delineated by starting weight, but women with weight loss were not identified as a separate group (but were considered of interest) (Figures S1 and S2). Therefore, In order to be able to use data from all the studies regardless of the number of time points and in order to explore risks with regard to weight loss as well as weight gain, we constructed the weight gain variables described below.

Construction of weight variables and contributing cohort studies

We constructed variables for weight at ages 18-24, 25-34, 35-44 and 45-54 years based on current weight or recalled weight at an age prior to enrolment. When weight at multiple time-points was available we used the earliest available weight within each age bracket. Weight change between age categories was computed by subtracting the later from the earlier weight. Annual rate of weight change was computed by the

difference in weight between the two age groups divided by the difference in the ages to which the weights applied.

Information on breast cancer characteristics

The main analytical endpoint was invasive or *in-situ* breast cancer diagnosed before the menopause. We conducted separate analyses by extent of breast cancer (in-situ vs invasive), oestrogen (ER)-receptor status (positive vs. negative), combined ER-receptor and progesterone (PR)-receptor status, and by clinicopathological surrogate definitions of luminal A and luminal B-like intrinsic breast cancer subtypes[3]. Sixteen studies provided information on in-situ and invasive breast cancer and one study (CSDLH) provided only information on invasive breast cancer. Immunohistochemistry data on estrogen (ER) status was available for 14 studies and all of ER and progesterone receptor (PR) status, as well as data on Human epidermal growth factor receptor-2 (HER2) oncogene expression were available for 10 studies. Given the absence of data on the proliferation marker KI-67, we adapted clinicopathological surrogate definitions of luminal A and luminal B-like intrinsic breast cancer subtypes proposed by the St Gallen Expert Consensus.[3] We classified all ER+PR+HER2- breast cancer as luminal A-like, ER+PR-HER2- and ER-PR+HER2- as luminal B-like HER2 negative, [ER+/PR+]HER2+ to luminal B-like HER2 positive, ER+PR- and ER-PR+ with HER2 status unknown as luminal B unclassified, ER+ or PR+ with other markers unknown as unclassified luminal, ER-PR-HER2- as triple-negative, ER-PR-HER2+ as HER2 enriched, ER- with PR unknown or PR- with ER unknown regardless of HER2 status as unclassified.

Statistical methods

Hazard ratios (HR) as estimates of relative risk of breast cancer were obtained from Cox proportional hazards models[4] with age as the underlying time-scale. All analyses were conducted using Stata 14.2 software[2]. Pooled analyses were adjusted for attained age (implicit in the Cox model) and cohort (including country within EPIC). In multivariable-adjusted models we additionally adjusted for year of birth (<1930, 1930-9, 1940-9, 1950-9, 1960-9, 1970-9, to ≥1980), age at menarche (7-11, 12-13, ≥14 years, not known), age at first birth (<25, 25-34, ≥35 years, nulliparous or parous but age not known), time since last birth (<5, 5-9, 10-14, 15-19, 20-24, 25-29, ≥30 years, nulliparous or parous but not known), parity (0, 1, 2, ≥3, parous but not known), adult height (in cms, continuous) and family history of breast cancer (yes, no, not known). In models additionally adjusted for starting weight, we fitted weight at this age as a continuous variable. Covariate information for all pregnancy-related variables and family history of breast cancer was time-updated, where possible. In sensitivity analyses, we investigated the effect of adjusting for cigarette smoking at recruitment (ever, never); alcohol consumption (<1, 1-2, 3-4, ≥5 drinks per week); physical activity level (cohort-specific quartiles); polycystic ovary syndrome (yes, no); childhood somatotype (3 groups); and history of mammographic screening (yes, no), among subjects with such data. Cohort studies with fewer than 10 cases for a weight change variable of interest were excluded from the model due to the difficulties in fitting models with very small numbers in cells. Linear trends per 5 kg weight gain were conducted based on continuous data.

In order to include the case-cohort study (CSDLH) in the pooled data set, we included Barlow weights[5] for CSDLH corresponding to a sampling fraction of 5.0 percent as an off-set in the model. We also applied Barlow weights with a sampling fraction of (effectively) 1.0 to all other cohorts using the stcasecoh command in Stata 14.2 software[2], which did not affect the results for those cohorts, but facilitated ease of obtaining results from a single pooled dataset. We tested for between-study heterogeneity in HRs using a two-stage model [6] and by fitting study as an interaction term in the pooled model. No appreciable between study-heterogeneity was observed. Tests for effect modification of weight change by starting weight, other breast cancer risk factors and by time since weight change were conducted with log likelihood ratio tests[7]. We estimated separate risk-factor associations by breast cancer type-specific outcomes with an augmentation method, in which HRs are obtained from a single model stratified on outcome type derived from a dataset in which records are repeated for each outcome [8].

Table S1: Study characteristics and numbers of premenopausal women with information on weight change, by cohort study

Study	Country	Age at recruitment, median	Number of subjects with available information on weight change between age categories											
			Ages 18-24 to 25-34		Ages 18-24 to 35-44		Ages 18-24 to 45-54		Ages 25-34 to 35-44		Ages 25-34 to 45-54		Ages 35-44 to 45-54	
			Cases	Subjects	Cases	Subjects	Cases	Subjects	Cases	Subjects	Cases	Subjects	Cases	Subjects
BWHS	USA	35.2	438	21239	672	34907	207	25153	336	18035	63	7039	173	19888
CLUE2	USA	36.5	32	1719	48	2873	22	2107	25	1286	0	0	13	1318
CSDLH (a)	Canada	42.6	58	155	134	550	19	378	0	0	0	0	0	0
CTS	USA	41.3	202	12019	387	23181	132	18032	64	4404	0	0	28	3968
E3N	France	42.5	0	0	0	0	0	0	0	0	0	0	599	27181
EPIC (b)	Europe	41.4	50	8609	277	20740	68	12822	0	0	0	0	0	0
GS	UK	38.8	104	20073	545	45954	235	23056	29	9074	0	0	238	23462
MCC	Australia	45.4	0	0	43	3671	53	6360	0	0	0	0	13	2081
NHS	USA	40.2	669	17164	1700	54003	1382	74092	719	19571	456	19501	1271	59971
NHS2	USA	34.9	1428	57230	2759	111456	1440	91930	1317	55012	584	44658	1454	92562
NOWAC	Norway	42.5	72	3858	595	43288	225	41944	52	3103	0	0	80	15123
NYUWHS	USA	39.5	0	0	0	0	0	0	0	0	0	0	57	2170
RERF	Japan	39.7	0	0	0	0	0	0	24	4595	0	0	0	0
SIS	USA	47.2	0	0	0	0	0	0	0	0	0	0	268	12791
SMC	Sweden	44.8	0	0	108	7823	106	10585	0	0	0	0	0	0
USRTC	USA	33.5	329	21816	384	34449	53	16928	267	20091	21	9279	34	15909
WLHS	Sweden	39.4	203	11813	386	27233	131	16821	60	5241	23	2596	23	4692
All		39.4	3585	175695	8038	410128	4073	340208	2893	140412	1147	83073	4251	281116

Abbreviations: BWHS: Black Women's Health Study; CLUE2: Campaign against Cancer and Heart Disease; CSDLH: Canadian Study of Diet, Lifestyle, and Health; CTS: California Teachers Study; E3N: Etude Epidémiologique auprès de femmes de la Mutuelle Générale de l'Education Nationale; EPIC: European Prospective Study into Cancer and Nutrition; GS Generations Study; MCC: Melbourne Collaborative Cohort Study; NHS: Nurses' Health Study; NHS2: Nurses' Health Study 2; NOWAC: Norwegian Women and Cancer Study; NYUWHS: New York University Women's Health Study; RERF: Radiation Effects Research Foundation Lifespan Study; SIS: Sister Study; SMC: Swedish Mammography Cohort; USRT: United States Radiologic Technologist Cohort; SWLHS: Sweden Women's Lifestyle and Health Study. References for details of each cohort have previously been published [9] (a) case-cohort selected from a cohort of 28000 women recruited under age 55 (b) EPIC includes cohorts from 5 European countries contributing to this analysis.

Table S2: Descriptive statistics on weight change between age categories in the pooled analysis

From age category, years	To age category, years	Number of contribu-ting cohorts	Median age in first age bracket	Median age in second age bracket	Median difference in age between categories, years	Median BMI at initial age group	Median change in weight, kg (b)	Median weight change, kg/year (b)	% subjects with weight gain	No. of subjects (a)	Median follow-up for breast cancer, years (c)	Number of incident cases
18-24	25-34	11	18.0	31.2	12.4	20.8	+4.5	+0.38	80.3	175695	17.2	3585
	35-44	13	18.0	38.8	20.0	20.7	+6.9	+0.35	86.1	410128	10.5	8038
	45-54	14	18.0	46.5	28.2	20.7	+10.0	+0.36	89.8	340208	4.2	4073
25-34	35-44	10	31.0	36.5	6.1	22.3	+3.2	+0.51	80.6	140412	12.0	2893
	45-54	5	31.7	46.1	14.3	22.2	+7.3	+0.50	90.4	83073	4.5	1147
35-44	45-54	13	38.7	46.2	8.1	22.9	+3.2	+0.46	82.4	281116	4.8	4251

(a) Subjects contribute to multiple weight change variables because the categories are not mutually exclusive
(b) Calculation includes women with weight loss as well as weight gain
(c) Follow-up starts at the age at the weight in the second age bracket and ends at breast cancer, death, age 55 or end of follow-up.

Table S3: Risk of premenopausal breast cancer in relation to weight change between various ages, by invasiveness of breast cancer

			Weight gain category, kg							Trend per 5kg weight gain (b)	
			Loss ≥5.0kg	± 4.9kg	Gain 5.0-9.9kg	Gain 10.0-14.9kg	Gain 15.0-19.9 kg	Gain ≥20.0kg			
Ages at weight change	Invasive-ness	No. of cases	HR (95% CI)(a)	HR (a)	HR (95% CI)(a)	HR (95% CI)(a)	HR (95% CI)(a)	HR (95% CI)(a)	P -int	HR (95% CI)(a)	P int-trend
Age 18-24 to 25-34	In situ	746	1.26 (0.93-1.71)	1.00 (ref)	0.91 (0.76-1.09)	0.82 (0.64-1.05)	0.83 (0.60-1.16)	0.70 (0.51-0.96)		0.93 (0.87-0.98)	
	Invasive	2588	1.06 (0.88-1.28)	1.00 (ref)	0.98 (0.89-1.08)	1.03 (0.91-1.17)	0.95 (0.80-1.13)	0.99 (0.85-1.16)	0.16	1.01 (0.98-1.03)	0.007
Age 18-24 to 35-44	In situ	1502	1.01 (0.78-1.30)	1.00 (ref)	0.91 (0.80-1.04)	0.82 (0.70-0.97)	0.84 (0.69-1.02)	0.74 (0.62-0.89)		0.95 (0.92-0.98)	
	Invasive	6134	0.90 (0.78-1.03)	1.00 (ref)	1.01 (0.94-1.07)	0.97 (0.89-1.04)	0.91 (0.83-1.00)	0.88 (0.80-0.96)	0.22	0.97 (0.96-0.99)	0.14
Age 18-24 to 45-54	In situ	781	0.90 (0.58-1.39)	1.00 (ref)	1.02 (0.83-1.24)	0.94 (0.76-1.17)	0.82 (0.63-1.06)	0.78 (0.63-0.97)		0.96 (0.93-1.00)	
	Invasive	3069	0.97 (0.78-1.21)	1.00 (ref)	0.96 (0.86-1.06)	0.97 (0.87-1.08)	0.87 (0.76-0.99)	0.81 (0.73-0.90)	0.96	0.96 (0.95-0.98)	0.96
Age 25-34 to 35-44	In situ	650	0.97 (0.65-1.45)	1.00 (ref)	1.00 (0.82-1.22)	0.92 (0.68-1.24)	0.64 (0.38-1.10)	0.77 (0.46-1.29)		0.92 (0.85-1.00)	
	Invasive	2012	0.96 (0.76-1.22)	1.00 (ref)	0.98 (0.87-1.10)	0.93 (0.78-1.11)	0.83 (0.64-1.09)	0.81 (0.61-1.09)	0.98	0.96 (0.92-1.01)	0.30
Age 25-34 to 45-54	In situ	279	1.14 (0.53-2.48)	1.00 (ref)	1.18 (0.88-1.57)	0.84 (0.58-1.22)	1.07 (0.69-1.66)	0.84 (0.53-1.34)		0.95 (0.87-1.03)	
	Invasive	760	1.24 (0.78-1.97)	1.00 (ref)	1.07 (0.89-1.27)	0.94 (0.75-1.16)	0.88 (0.66-1.17)	0.98 (0.75-1.29)	0.85	0.97 (0.92-1.02)	0.70
Age 35-44 to 45-54	In situ	865	1.22 (0.86-1.72)	1.00 (ref)	1.04 (0.88-1.24)	0.94 (0.73-1.21)	1.29 (0.93-1.79)	1.21 (0.85-1.71)		1.02 (0.97-1.08)	
	Invasive	3140	1.11 (0.92-1.35)	1.00 (ref)	0.96 (0.87-1.05)	1.04 (0.91-1.18)	1.04 (0.86-1.26)	0.86 (0.69-1.06)	0.39	0.98 (0.95-1.01)	0.18

Abbreviations: CI, confidence interval; ER, oestrogen-receptor; HR, hazard ratio; PR, progesterone-receptor
(a) HRs are adjusted for attained age, cohort, year of birth, adult height, weight at start of age range, age at menarche, age at first birth, number of births, time since last birth and family history of breast cancer
(b) Including subjects with weight gain only

Table S4: Risk of premenopausal breast cancer in relation to weight change between various ages, by oestrogen and progesterone-receptor status of the breast cancer

			Weight gain category, kg								
			Loss ≥5.0kg	± 4.9kg	Gain 5.0-9.9kg	Gain 10.0-14.9kg	Gain 15.0-19.9kg	Gain ≥20.0kg		Trend per 5kg gain (b)	
Ages at weight change	ER and PR status	No. of cases	HR (95% CI)(a)	HR (a)	HR (95% CI)(a)	HR (95% CI)(a)	HR (95% CI)(a)	HR (95% CI)(a)	P -int	HR (95% CI) (a)	P int-trend
Ages 18-24 to 25-34	ER+PR+	1517	0.93 (0.72-1.19)	1.00 (ref)	0.90 (0.79-1.03)	0.93 (0.79-1.09)	0.79 (0.63-1.00)	0.85 (0.70-1.04)		0.97 (0.93-1.00)	
	ER+PR-	221	1.23 (0.70-2.16)	1.00 (ref)	0.70 (0.48-1.02)	1.09 (0.73-1.64)	1.09 (0.64-1.84)	0.95 (0.58-1.56)		1.04 (0.95-1.14)	
	ER-PR+	88	1.72 (0.81-3.67)	1.00 (ref)	0.78 (0.45-1.38)	1.23 (0.67-2.25)	0.65 (0.23-1.83)	0.24 (0.06-1.00)		0.87 (0.74-1.03)	
	ER-PR-	491	1.11 (0.73-1.69)	1.00 (ref)	1.13 (0.90-1.42)	1.04 (0.78-1.39)	0.90 (0.60-1.34)	1.26 (0.93-1.72)	0.20	1.03 (0.97-1.08)	0.07
Ages 18-24 to 35-44	ER+PR+	3118	0.86 (0.71-1.05)	1.00 (ref)	0.98 (0.89-1.07)	0.97 (0.87-1.08)	0.89 (0.78-1.02)	0.78 (0.69-0.88)		0.96 (0.94-0.98)	
	ER+PR-	484	1.10 (0.72-1.68)	1.00 (ref)	0.89 (0.70-1.13)	0.93 (0.71-1.21)	0.88 (0.63-1.23)	0.62 (0.45-0.86)		0.92 (0.87-0.98)	
	ER-PR+	198	1.03 (0.49-2.14)	1.00 (ref)	1.13 (0.79-1.63)	1.24 (0.83-1.86)	1.12 (0.68-1.85)	0.51 (0.29-0.90)		0.95 (0.87-1.03)	
	ER-PR-	1001	0.86 (0.60-1.23)	1.00 (ref)	1.06 (0.90-1.26)	1.06 (0.88-1.29)	1.06 (0.84-1.34)	1.10 (0.91-1.34)	0.10	1.01 (0.98-1.05)	0.005
Ages 18-24 to 45-54	ER+PR+	1774	0.69 (0.49-0.96)	1.00 (ref)	0.99 (0.87-1.14)	1.00 (0.86-1.15)	0.87 (0.73-1.03)	0.85 (0.74-0.97)		0.97 (0.95-0.99)	
	ER+PR-	289	1.02 (0.52-1.97)	1.00 (ref)	0.96 (0.70-1.34)	1.03 (0.73-1.44)	0.84 (0.56-1.27)	0.59 (0.40-0.85)		0.90 (0.85-0.96)	
	ER-PR+	107	1.92 (0.79-4.68)	1.00 (ref)	1.14 (0.66-1.97)	1.13 (0.64-2.01)	0.39 (0.15-1.03)	0.96 (0.55-1.68)		0.98 (0.89-1.07)	
	ER-PR-	496	1.23 (0.72-2.08)	1.00 (ref)	1.30 (1.00-1.69)	1.18 (0.89-1.57)	1.12 (0.82-1.55)	1.06 (0.81-1.38)	0.20	0.99 (0.95-1.03)	0.08
Age 25-34 to 35-44	ER+PR+	1308	0.99 (0.75-1.31)	1.00 (ref)	0.99 (0.85-1.15)	1.05 (0.85-1.30)	0.75 (0.52-1.07)	0.73 (0.49-1.07)		0.95 (0.90-1.00)	
	ER+PR-	176	1.14 (0.57-2.25)	1.00 (ref)	0.98 (0.66-1.46)	0.82 (0.44-1.51)	0.69 (0.25-1.89)	0.58 (0.18-1.83)		0.88 (0.76-1.02)	
	ER-PR+	73	0.84 (0.26-2.70)	1.00 (ref)	0.99 (0.55-1.79)	0.33 (0.08-1.35)	0.00 (0.00-0.00)	0.43 (0.06-3.08)		0.81 (0.57-1.14)	
	ER-PR-	384	0.67 (0.37-1.23)	1.00 (ref)	1.08 (0.83-1.40)	1.25 (0.88-1.78)	1.05 (0.60-1.83)	1.23 (0.71-2.13)	0.73	1.06 (0.98-1.13)	0.03
Age 25-34 to 45-54	ER+PR+	637	1.49 (0.94-2.38)	1.00 (ref)	1.17 (0.96-1.41)	0.93 (0.73-1.18)	0.88 (0.64-1.21)	1.00 (0.75-1.34)		0.96 (0.91-1.01)	
	ER+PR-	79	0.92 (0.22-3.88)	1.00 (ref)	0.79 (0.46-1.37)	0.85 (0.45-1.60)	0.73 (0.31-1.74)	0.32 (0.10-1.05)		0.85 (0.72-1.01)	
	ER-PR+	33	0.00 (0.00-0.00)	1.00 (ref)	0.99 (0.44-2.24)	0.49 (0.14-1.71)	0.92 (0.27-3.20)	0.81 (0.23-2.84)		0.87 (0.69-1.09)	
	ER-PR-	135	0.31 (0.04-2.28)	1.00 (ref)	1.06 (0.70-1.61)	1.02 (0.62-1.67)	0.99 (0.53-1.86)	0.65 (0.32-1.34)	0.81	0.97 (0.87-1.08)	0.49
Age 35-44 to 45-54	ER+PR+	2058	1.06 (0.84-1.35)	1.00 (ref)	0.97 (0.87-1.09)	1.09 (0.94-1.28)	1.20 (0.97-1.49)	0.94 (0.74-1.20)		1.00 (0.97-1.04)	
	ER+PR-	335	1.22 (0.72-2.06)	1.00 (ref)	0.87 (0.65-1.15)	0.94 (0.63-1.38)	1.13 (0.67-1.92)	0.62 (0.30-1.26)		0.94 (0.85-1.04)	
	ER-PR+	122	0.61 (0.19-1.93)	1.00 (ref)	0.68 (0.41-1.11)	0.83 (0.43-1.60)	1.17 (0.51-2.69)	0.81 (0.30-2.20)		0.96 (0.81-1.13)	
	ER-PR-	529	0.85 (0.51-1.41)	1.00 (ref)	1.14 (0.92-1.40)	1.09 (0.81-1.47)	0.60 (0.34-1.07)	0.88 (0.54-1.43)	0.51	0.98 (0.90-1.06)	0.62

Abbreviations: CI, confidence interval; ER, oestrogen-receptor; HR, hazard ratio; PR, progesterone-receptor
(a) HRs are adjusted for attained age, cohort, year of birth, adult height, weight at start of age range, age at menarche, age at first birth, number of births, time since last birth and family history of breast cancer
(b) Including subjects with weight gain only

Table S5: Risk of premenopausal breast cancer in relation to weight change between various ages, by initial BMI

Age and initial BMI (kg/m^2)	Age period of weight gain	No. of cases	Loss ≥5.0 kg HR (95% CI)(a)	± 4.9 kg HR (a)	Gain 5.0-9.9 kg HR (95% CI)(a)	Gain 10.0-14.9 kg HR (95% CI)(a)	Gain 15.0-19.9 kg HR (95% CI)(a)	Gain ≥20.0 kg HR (95% CI)(a)	P - int	Trend per 5 kg weight gain (b) HR (95% CI) (a)	P int-trend
BMI at ages 18-24	Age 18-24 to 25-34										
<18.5		566	1.29 (0.18-9.38)	1.00 (ref)	1.02 (0.84-1.25)	1.09 (0.86-1.38)	1.22 (0.89-1.68)	1.05 (0.73-1.51)	0.66	1.02 (0.96-1.08)	0.41
18.5-24.9		2752	1.07 (0.88-1.30)	1.00 (ref)	0.94 (0.85-1.04)	0.95 (0.84-1.08)	0.84 (0.71-1.01)	0.85 (0.72-1.00)		0.97 (0.95-1.00)	
≥25		267	1.23 (0.88-1.72)	1.00 (ref)	1.25 (0.82-1.89)	0.89 (0.54-1.47)	1.22 (0.75-1.98)	1.01 (0.69-1.48)		1.00 (0.94-1.06)	
BMI at ages 18-24	Age 18-24 to 35-44										
<18.5		1279	-	1.00 (ref)	1.06 (0.91-1.23)	1.04 (0.89-1.22)	0.91 (0.74-1.11)	0.90 (0.74-1.10)	0.13	0.99 (0.96-1.02)	0.40
18.5-24.9		6185	0.81 (0.69-0.95)	1.00 (ref)	0.96 (0.90-1.03)	0.90 (0.84-0.98)	0.88 (0.79-0.97)	0.84 (0.77-0.91)		0.97 (0.95-0.98)	
≥25		574	1.30 (1.02-1.66)	1.00 (ref)	1.21 (0.89-1.64)	1.04 (0.75-1.45)	1.26 (0.91-1.73)	1.06 (0.82-1.36)		0.98 (0.94-1.02)	
BMI at ages 18-24	Age 18-24 to 45-54										
<18.5		614	-	1.00 (ref)	1.00 (0.77-1.30)	0.93 (0.72-1.21)	0.84 (0.63-1.12)	0.62 (0.47-0.83)	0.54	0.91 (0.87-0.96)	0.02
18.5-24.9		3155	0.99 (0.77-1.28)	1.00 (ref)	0.95 (0.86-1.05)	0.95 (0.85-1.06)	0.87 (0.77-0.99)	0.83 (0.75-0.93)		0.98 (0.96-0.99)	
≥25		304	0.96 (0.68-1.37)	1.00 (ref)	1.09 (0.73-1.64)	1.17 (0.78-1.75)	0.79 (0.49-1.28)	0.84 (0.61-1.16)		0.94 (0.89-0.99)	
BMI at ages 25-34	Age 25-34 to 35-44										
<18.5		138	-	1.00 (ref)	1.37 (0.90-2.08)	1.56 (0.78-3.10)	3.97 (1.73-9.09)	2.25 (0.71-7.12)	0.10	1.27 (1.10-1.47)	0.006
18.5-24.9		2120	1.10 (0.83-1.46)	1.00 (ref)	0.94 (0.84-1.06)	0.85 (0.71-1.02)	0.71 (0.52-0.98)	0.79 (0.56-1.12)		0.93 (0.89-0.98)	
25-29.9		447	0.94 (0.67-1.32)	1.00 (ref)	1.04 (0.81-1.33)	1.22 (0.92-1.62)	0.91 (0.60-1.39)	0.86 (0.57-1.31)		0.98 (0.91-1.05)	
≥30		188	0.94 (0.62-1.43)	1.00 (ref)	0.82 (0.54-1.25)	0.88 (0.55-1.42)	0.79 (0.42-1.49)	0.94 (0.54-1.63)		0.98 (0.88-1.10)	
BMI at ages 25-34	Age 25-34 to 45-54										
<18.5		46	-	1.00 (ref)	1.87 (0.95-3.68)	1.86 (0.76-4.57)	2.11 (0.61-7.30)	2.47 (0.56-10.8)	0.22	1.22 (0.98-1.52)	0.30
18.5-24.9		886	1.82 (1.12-2.98)	1.00 (ref)	1.14 (0.98-1.34)	0.87 (0.71-1.07)	0.85 (0.64-1.12)	0.98 (0.74-1.29)		0.97 (0.93-1.02)	

25-29.9		147	0.77 (0.35-1.72)	1.00 (ref)	0.66 (0.39-1.10)	0.93 (0.58-1.49)	0.75 (0.43-1.32)	0.81 (0.50-1.30)		0.96 (0.88-1.05)	
≥30		68	1.26 (0.54-2.95)	1.00 (ref)	1.05 (0.46-2.39)	1.50 (0.70-3.19)	1.42 (0.61-3.32)	1.09 (0.51-2.36)		0.95 (0.83-1.09)	
BMI at ages 35-44	Age 35-44 to 45-54										
<18.5		148	3.43 (0.48-24.6)	1.00 (ref)	1.04 (0.69-1.58)	1.24 (0.63-2.46)	1.10 (0.35-3.47)	0.00 (0.00-0.00)	0.60	1.01 (0.84-1.21)	0.58
18.5-24.9		3069	1.20 (0.91-1.58)	1.00 (ref)	0.97 (0.88-1.06)	1.04 (0.90-1.19)	0.93 (0.74-1.17)	0.95 (0.74-1.22)		0.98 (0.94-1.02)	
25-29.9		700	1.23 (0.93-1.62)	1.00 (ref)	1.03 (0.85-1.26)	1.12 (0.89-1.41)	1.41 (1.07-1.87)	1.04 (0.77-1.42)		1.02 (0.97-1.08)	
≥30		334	0.90 (0.65-1.25)	1.00 (ref)	0.85 (0.62-1.16)	1.03 (0.74-1.44)	1.02 (0.67-1.57)	0.74 (0.47-1.17)		0.97 (0.89-1.05)	

Abbreviations: HR, hazard ratio; CI, confidence interval

(a) HRs are adjusted for attained age, cohort, year of birth, adult height, starting weight, age at menarche, age at first birth, number of births, time since last birth and family history of breast cancer

(b) Including subjects with weight gain only

Table S6: Risk of premenopausal breast cancer per 5 kg weight gain, by age at weight change and other breast cancer risk factors

	Age at weight change, years											
	Age 18-24 to 25-34		Age 18-24 to 35-44		Age 18-24 to 45-54		Age 25-34 to 35-44		Age 25-34 to 45-54		Age 35-44 to 45-54	
Variable (a)	No. cases	HR (95% CI) (b)	No. cases	HR (95% CI) (b)	No. cases	HR (95% CI) (b)	No. cases	HR (95% CI) (b)	No. cases	HR (95% CI) (b)	No. cases	HR (95% CI) (b)
Adult height, cms												
<160	490	1.00 (0.94-1.06)	1174	0.97 (0.94-1.01)	637	0.95 (0.91-0.99)	412	1.00 (0.91-1.09)	193	0.96 (0.86-1.06)	669	1.01 (0.94-1.08)
160-169	1528	0.96 (0.93-1.00)	3762	0.96 (0.95-0.98)	1997	0.96 (0.94-0.98)	1224	0.96 (0.91-1.01)	550	0.96 (0.91-1.02)	1956	0.99 (0.95-1.03)
≥170	903	1.00 (0.97-1.04)	2018	0.98 (0.96-1.00)	1019	0.98 (0.95-1.00)	691	0.96 (0.91-1.02)	299	0.99 (0.93-1.06)	930	0.98 (0.93-1.03)
BMI at age 18-24 yrs, kg/m^2		P int=0.20		P int=0.54		P int=0.41		P int=0.75		P int=0.80		P int=0.81
<18.5	550	1.02 (0.96-1.08)	1252	0.99 (0.96-1.02)	606	0.91 (0.87-0.96)	341	1.13 (1.03-1.25)	137	0.96 (0.84-1.10)	403	0.85 (0.76-0.95)
18.4-24.9	2208	0.97 (0.95-1.00)	5331	0.97 (0.95-0.98)	2826	0.98 (0.96-0.99)	1726	0.96 (0.91-1.00)	755	0.99 (0.94-1.04)	2077	1.01 (0.97-1.05)
25.0-29.9	163	1.00 (0.94-1.06)	371	0.98 (0.94-1.02)	221	0.94 (0.89-0.99)	156	0.91 (0.82-1.00)	74	0.86 (0.76-0.97)	190	0.98 (0.90-1.06)
		P int=0.41		P int=0.40		P int=0.02		P int=0.01		P int=0.20		P int=0.12
Childhood shape (c)												
1-3	1202	0.99 (0.95-1.03)	2812	0.96 (0.94-0.99)	1890	0.96 (0.94-0.98)	1120	0.97 (0.91-1.03)	643	0.96 (0.91-1.02)	2075	1.00 (0.96-1.04)
4-6	321	0.98 (0.93-1.04)	711	0.98 (0.94-1.01)	450	0.98 (0.95-1.02)	329	0.94 (0.86-1.02)	179	0.90 (0.82-0.98)	448	0.98 (0.92-1.05)
7-9	7	0.92 (0.63-1.35)	13	1.00 (0.80-1.26)	9	1.13 (0.92-1.39)	6	0.61 (0.25-1.48)	3	0.98 (0.61-1.58)	11	1.21 (0.93-1.56)
Childhood weight relative to peers		P int=0.93		P int=0.80		P int=0.19		P int=0.40		P int=0.39		P int=0.37
Lighter	101	0.94 (0.80-1.12)	379	0.93 (0.86-1.00)	138	0.91 (0.82-1.02)	37	0.89 (0.62-1.28)	8	0.53 (0.20-1.38)	154	1.07 (0.97-1.19)
Similar	153	0.92 (0.80-1.04)	640	0.99 (0.94-1.04)	280	0.97 (0.91-1.04)	53	1.04 (0.82-1.31)	12	1.02 (0.64-1.64)	242	0.97 (0.88-1.06)
Heavier	23	1.13 (0.91-1.40)	132	0.86 (0.78-0.96)	61	0.96 (0.84-1.08)	8	1.00 (0.58-1.70)	3	1.05 (0.45-2.46)	80	0.96 (0.84-1.09)
		P int=0.28		P int=0.06		P int=0.63		P int=0.92		P int=0.18		P int=0.42
Age at menarche, yrs												
<12	1683	0.96 (0.93-1.00)	3837	0.97 (0.95-0.98)	2045	0.95 (0.93-0.98)	1327	0.96 (0.91-1.00)	575	0.98 (0.93-1.04)	1987	0.99 (0.95-1.03)
12-13	508	0.97 (0.91-1.04)	1420	0.94 (0.91-0.98)	745	0.96 (0.92-0.99)	411	0.99 (0.90-1.08)	192	0.97 (0.87-1.07)	686	1.00 (0.93-1.07)
≥14	704	1.01 (0.97-1.05)	1574	0.98 (0.96-1.01)	804	0.99 (0.96-1.02)	571	0.97 (0.91-1.04)	266	0.97 (0.90-1.05)	830	0.99 (0.94-1.04)
		P int=0.15		P int=0.11		P int=0.14		P int=0.83		P int=0.96		P int=0.97

Ever having had a birth												
No	514	0.95 (0.90-1.00)	1062	0.96 (0.93-0.99)	499	0.95 (0.91-0.99)	423	0.93 (0.86-1.01)	156	0.93 (0.84-1.02)	477	0.98 (0.92-1.05)
Yes	2295	0.99 (0.96-1.02)	5758	0.97 (0.96-0.99)	3125	0.97 (0.95-0.98)	1887	0.98 (0.94-1.02)	882	0.98 (0.94-1.03)	2988	0.99 (0.96-1.02)
Age at first birth, yrs (parous)		P int=0.11		P int=0.50		P int=0.43		P int=0.30		P int=0.27		P int=0.84
<25	712	0.99 (0.95-1.03)	2202	0.98 (0.96-1.00)	1313	0.98 (0.95-1.00)	583	1.02 (0.95-1.09)	331	1.01 (0.94-1.08)	1262	0.98 (0.94-1.03)
25-34	1427	0.98 (0.95-1.02)	3132	0.96 (0.94-0.98)	1611	0.96 (0.93-0.98)	1150	0.96 (0.90-1.01)	488	0.98 (0.92-1.04)	1524	1.00 (0.96-1.04)
≥35	139	1.02 (0.92-1.14)	391	0.98 (0.92-1.03)	188	0.99 (0.92-1.05)	145	0.94 (0.82-1.07)	63	0.90 (0.76-1.05)	199	0.96 (0.86-1.08)
Number of births (parous)		P int=0.79		P int=0.44		P int=0.62		P int=0.33		P int=0.37		P int=0.78
1	500	0.96 (0.91-1.02)	985	0.98 (0.95-1.01)	459	1.00 (0.97-1.04)	361	0.93 (0.85-1.01)	134	0.99 (0.89-1.09)	473	1.01 (0.94-1.08)
2	1116	0.99 (0.96-1.03)	2789	0.97 (0.95-0.99)	1409	0.97 (0.94-0.99)	921	1.00 (0.94-1.06)	424	1.00 (0.94-1.07)	1395	1.00 (0.96-1.05)
≥3	661	1.00 (0.95-1.05)	1956	0.97 (0.94-0.99)	1247	0.95 (0.92-0.98)	587	0.96 (0.89-1.04)	324	0.95 (0.88-1.03)	1116	0.96 (0.91-1.01)
Family history of breast cancer		P int=0.61		P int=0.75		P int=0.07		P int=0.30		P int=0.59		P int=0.39
No	2226	0.97 (0.95-1.00)	5158	0.97 (0.95-0.98)	2713	0.96 (0.95-0.98)	1739	0.98 (0.94-1.02)	736	1.00 (0.95-1.04)	2404	0.99 (0.96-1.03)
Yes	649	1.00 (0.95-1.05)	1548	0.97 (0.95-1.00)	874	0.97 (0.94-1.00)	571	0.94 (0.87-1.01)	306	0.91 (0.84-0.99)	1057	0.98 (0.94-1.02)
		P int=0.31		P int=0.71		P int=0.78		P int=0.31		P int=0.05		P int=0.59
Ethnicity												
Caucasian	2244	0.97 (0.94-1.00)	5265	0.96 (0.95-0.98)	3045	0.96 (0.95-0.98)	1882	0.96 (0.92-1.00)	933	0.96 (0.92-1.00)	2686	0.99 (0.96-1.02)
Black	441	1.00 (0.95-1.04)	723	0.99 (0.96-1.02)	233	0.97 (0.93-1.02)	295	1.00 (0.92-1.09)	67	1.10 (0.96-1.25)	193	0.98 (0.89-1.08)
Asian	53	1.04 (0.83-1.30)	80	1.03 (0.87-1.22)	46	0.87 (0.70-1.08)	48	0.72 (0.48-1.07)	16	0.81 (0.48-1.38)	39	0.86 (0.55-1.35)
		P int=0.52		P int=0.30		P int=0.60		P int=0.19		P int=0.13		P int=0.82

Abbreviations: BMI, Body-mass Index; CI, confidence interval; HR, hazard ratio

(a) Stratifying variables are time-updated, where possible, for anthropometric and reproductive variables and family history of breast cancer.

(b) Hazard ratio represents linear trend per 5 kg weight gain fitted across weight values ≥0.0 kg and are adjusted for attained age and cohort, year of birth, age at menarche, age at first birth, number of births, time since last birth, family history of breast cancer, adult height and weight at the start of each weight change age group.

(c) Childhood shape categorised according to pictogram scale, with 1-3 representing the leanest body shapes and 7-9 representing the heaviest body shapes.

Table S7: Risk of premenopausal breast cancer in relation to weight change category, by age at weight change and time since weight change

	Time since weight change, years								
	<5		5-9		10-14		≥15		p-inter-action
Age at weight change and weight change category, kg	No. of cases	HR (95% CI) (a)	No. of cases	HR (95% CI) (a)	No. of cases	HR (95% CI) (a)	No. of cases	HR (95% CI) (a)	
Weight change from 18-24 to 25-34 years									
Lost ≥5.0	14	1.02 (0.58-1.76)	32	1.02 (0.70-1.47)	69	1.17 (0.90-1.51)	80	1.07 (0.85-1.36)	
+-4.9	132	1.00 (ref)	303	1.00 (ref)	574	1.00 (ref)	721	1.00 (ref)	
5.0-9.9	90	1.35 (1.03-1.77)	167	1.09 (0.91-1.32)	225	0.80 (0.68-0.93)	307	0.96 (0.84-1.10)	
10.0-14.9	44	1.23 (0.88-1.74)	103	1.26 (1.01-1.58)	125	0.83 (0.68-1.01)	140	0.90 (0.75-1.07)	
15.0-19.9	11	0.59 (0.32-1.09)	46	1.06 (0.78-1.45)	67	0.84 (0.65-1.09)	81	1.05 (0.83-1.32)	
≥20.0	22	0.88 (0.56-1.39)	48	0.84 (0.62-1.15)	98	0.93 (0.75-1.16)	86	0.89 (0.71-1.11)	0.01
Trend per 5 kg weight gain (b)	253	0.99 (0.92-1.06)	573	1.00 (0.96-1.05)	955	0.98 (0.94-1.02)	1140	0.98 (0.94-1.02)	0.84
Weight change from 18-24 to 35-44 years									
Lost ≥5.0	89	1.01 (0.81-1.26)	106	0.78 (0.64-0.96)	95	1.09 (0.88-1.35)	21	0.76 (0.49-1.20)	
+-4.9	768	1.00 (ref)	1181	1.00 (ref)	795	1.00 (ref)	237	1.00 (ref)	
5.0-9.9	579	1.07 (0.96-1.19)	764	0.94 (0.85-1.03)	477	0.95 (0.85-1.06)	125	1.01 (0.81-1.25)	
10.0-14.9	343	0.93 (0.82-1.06)	465	0.88 (0.79-0.98)	311	1.01 (0.89-1.16)	59	0.85 (0.64-1.13)	
15.0-19.9	195	0.89 (0.76-1.04)	256	0.82 (0.72-0.94)	170	0.97 (0.82-1.14)	38	1.08 (0.77-1.52)	
≥20.0	322	0.95 (0.83-1.08)	384	0.80 (0.71-0.90)	218	0.80 (0.69-0.93)	40	0.81 (0.58-1.14)	0.22
Trend per 5 kg weight gain (b)	2006	0.99 (0.97-1.01)	2744	0.96 (0.94-0.98)	1774	0.96 (0.94-0.99)	430	0.97 (0.91-1.02)	0.25
Weight change from 18-24 to 45-54 years									
Lost ≥5.0	97	1.02 (0.82-1.26)	27	0.77 (0.52-1.15)					
+-4.9	796	1.00 (ref)	296	1.00 (ref)					
5.0-9.9	668	0.96 (0.87-1.07)	230	0.98 (0.83-1.17)					
10.0-14.9	562	0.96 (0.86-1.07)	183	0.97 (0.81-1.17)					
15.0-19.9	336	0.85 (0.75-0.97)	109	0.90 (0.72-1.12)					
≥20.0	592	0.79 (0.71-0.88)	177	0.83 (0.68-1.00)					0.83
Trend per 5 kg weight gain (b)	2721	0.97 (0.95-0.98)	932	0.96 (0.93-0.99)					0.78

Weight change from 25-34 to 35-44 years									
Lost ≥5.0	28	1.00 (0.68-1.47)	40	0.99 (0.71-1.37)	37	1.01 (0.72-1.42)	14	1.01 (0.59-1.73)	
+-4.9	421	1.00 (ref)	634	1.00 (ref)	569	1.00 (ref)	250	1.00 (ref)	
5.0-9.9	156	1.02 (0.85-1.22)	191	0.93 (0.79-1.10)	134	0.96 (0.80-1.17)	42	1.00 (0.72-1.39)	
10.0-14.9	68	0.97 (0.75-1.26)	91	1.01 (0.81-1.26)	47	0.85 (0.63-1.15)	14	1.03 (0.60-1.77)	
15.0-19.9	27	0.86 (0.58-1.28)	33	0.84 (0.59-1.19)	19	0.87 (0.55-1.38)	2	0.41 (0.10-1.65)	
≥20.0	33	1.08 (0.75-1.55)	33	0.91 (0.64-1.29)	9	0.51 (0.26-0.98)	1	0.34 (0.05-2.40)	0.91
Trend per 5 kg weight gain (b)	616	0.98 (0.92-1.04)	826	0.98 (0.93-1.04)	641	0.92 (0.85-0.99)	244	0.96 (0.83-1.12)	0.52
Weight change from 25-34 to 45-54 years									
Lost ≥5.0	27	1.41 (0.94-2.12)	6	1.01 (0.44-2.29)					
+-4.9	295	1.00 (ref)	122	1.00 (ref)					
5.0-9.9	232	1.08 (0.91-1.29)	101	1.20 (0.92-1.56)					
10.0-14.9	121	0.91 (0.74-1.13)	51	1.07 (0.77-1.48)					
15.0-19.9	67	0.93 (0.71-1.21)	20	0.84 (0.52-1.35)					
≥20.0	79	0.96 (0.74-1.24)	26	1.04 (0.68-1.59)					0.75
Trend per 5 kg weight gain (b)	742	0.97 (0.93-1.02)	300	0.98 (0.91-1.06)					0.85
Weight change from 35-44 to 45-54 years									
Lost ≥5.0	127	1.14 (0.95-1.37)	37	1.03 (0.74-1.43)					
+-4.9	1810	1.00 (ref)	730	1.00 (ref)					
5.0-9.9	629	0.97 (0.88-1.06)	229	0.99 (0.85-1.15)					
10.0-14.9	290	1.05 (0.92-1.19)	98	1.08 (0.87-1.33)					
15.0-19.9	128	1.09 (0.91-1.30)	38	1.06 (0.77-1.48)					
≥20.0	105	0.91 (0.74-1.11)	30	0.99 (0.68-1.43)					0.99
Trend per 5 kg weight gain (b)	2582	0.99 (0.96-1.02)	973	1.00 (0.95-1.06)					0.88

Abbreviations: CI, confidence interval; HR, hazard ratio

(a) HRs adjusted for attained age and study cohort, year of birth, age at menarche, age at first birth, number of births, time since last birth, family history of breast cancer, adult height and weight at the start of each weight change age group

(b) Linear trend per 5kg weight gain fitted across weight change values from ≥0.0 kg

Table S8: Results from selected sensitivity analyses: Risk of premenopausal breast cancer in relation to weight change category, by age at weight change. For (1) all subjects included in main analysis (2) adjusting additionally for number of years between weight assessments (3) excluding first two years of follow-up (4) strictly known premenopausal time only (5) adjusting for weight at ages 18-24 in subset of women with available data.

	All subjects		Adjusting additionally for the number of years between weight assessments		Analyses excluding first two years of follow-up		Analyses restricted to strictly known premenopausal follow-up time only		Analyses adjusted for weight at ages 18-24 years in subset of women with available data	
Age at weight change and weight change category, kg	No. of cases	HR (95% CI) (a)	No. of cases	HR (95% CI) (b)	No. of cases	HR (95% CI) (a)	No. of cases	HR (95% CI) (a)	No. of cases	HR (95% CI) (c)
Weight change from 18-24 to 25-34 years										
Lost ≥5.0	195	1.09 (0.93-1.27)	195	1.09 (0.93-1.28)	190	1.09 (0.93-1.28)	129	1.11 (0.91-1.35)	195	1.09 (0.93-1.27)
+-4.9	1730	1.00 (ref)	1730	1.00 (ref)	1690	1.00 (ref)	1092	1.00 (ref)	1730	1.00 (ref)
5.0-9.9	789	0.96 (0.88-1.04)	789	0.96 (0.88-1.05)	755	0.94 (0.86-1.03)	480	0.99 (0.89-1.10)	789	0.96 (0.88-1.04)
10.0-14.9	412	0.97 (0.87-1.08)	412	0.97 (0.87-1.08)	399	0.97 (0.86-1.08)	229	0.98 (0.85-1.13)	412	0.97 (0.87-1.08)
15.0-19.9	205	0.93 (0.80-1.08)	205	0.94 (0.81-1.09)	201	0.94 (0.81-1.10)	98	0.88 (0.71-1.08)	205	0.93 (0.80-1.08)
≥20.0	254	0.88 (0.77-1.02)	254	0.89 (0.78-1.02)	249	0.90 (0.78-1.04)	111	0.85 (0.70-1.04)	254	0.88 (0.77-1.02)
Trend per 5 kg gain (d)	2921	0.98 (0.96-1.01)	2921	0.98 (0.96-1.01)	2847	0.98 (0.96-1.01)	1703	0.98 (0.95-1.01)	2921	0.98 (0.96-1.01)
Weight change from 18-24 to 35-44 years										
Lost ≥5.0	311	0.92 (0.81-1.04)	311	0.92 (0.81-1.04)	286	0.92 (0.81-1.04)	209	0.94 (0.81-1.10)	311	0.92 (0.81-1.04)
+-4.9	2981	1.00 (ref)	2981	1.00 (ref)	2734	1.00 (ref)	2025	1.00 (ref)	2981	1.00 (ref)
5.0-9.9	1945	0.98 (0.93-1.04)	1945	0.98 (0.92-1.04)	1756	0.97 (0.92-1.03)	1218	0.97 (0.90-1.04)	1945	0.98 (0.93-1.04)
10.0-14.9	1178	0.93 (0.87-0.99)	1178	0.92 (0.86-0.99)	1068	0.92 (0.86-0.99)	728	0.95 (0.87-1.04)	1178	0.93 (0.87-0.99)
15.0-19.9	659	0.89 (0.82-0.97)	659	0.89 (0.81-0.97)	606	0.90 (0.82-0.98)	390	0.93 (0.84-1.04)	659	0.89 (0.82-0.97)
≥20.0	964	0.85 (0.79-0.92)	964	0.84 (0.78-0.91)	881	0.84 (0.78-0.91)	462	0.78 (0.70-0.86)	964	0.85 (0.79-0.92)
Trend per 5 kg gain (d)	6954	0.97 (0.96-0.98)	6954	0.97 (0.96-0.98)	6336	0.97 (0.96-0.98)	4305	0.96 (0.94-0.98)	6954	0.97 (0.96-0.98)
Weight change from 18-24 to 45-54 years										
Lost ≥5.0	124	0.95 (0.78-1.15)	124	0.95 (0.78-1.15)	99	0.91 (0.73-1.13)	94	1.01 (0.81-1.26)	124	0.95 (0.78-1.15)
+-4.9	1092	1.00 (ref)	1092	1.00 (ref)	935	1.00 (ref)	838	1.00 (ref)	1092	1.00 (ref)
5.0-9.9	898	0.97 (0.89-1.06)	898	0.97 (0.89-1.06)	772	0.98 (0.89-1.08)	703	1.02 (0.92-1.12)	898	0.97 (0.89-1.06)
10.0-14.9	745	0.96 (0.87-1.06)	745	0.96 (0.88-1.06)	637	0.96 (0.86-1.06)	585	1.03 (0.92-1.14)	745	0.96 (0.87-1.06)
15.0-19.9	445	0.86 (0.77-0.96)	445	0.86 (0.77-0.97)	382	0.85 (0.75-0.96)	330	0.88 (0.78-1.01)	445	0.86 (0.77-0.96)

≥20.0	769	0.80 (0.73-0.88)	769	0.80 (0.73-0.88)	709	0.81 (0.73-0.90)	553	0.82 (0.74-0.92)	769	0.80 (0.73-0.88)
Trend per 5 kg gain (d)	3653	0.96 (0.95-0.98)	3653	0.96 (0.95-0.98)	3190	0.97 (0.95-0.98)	2793	0.96 (0.95-0.98)	3653	0.96 (0.95-0.98)
Weight change from 25-34 to 35-44 years										
Lost ≥5.0	119	1.00 (0.83-1.21)	119	1.00 (0.82-1.21)	118	1.00 (0.82-1.21)	92	1.12 (0.90-1.39)	116	0.97 (0.80-1.17)
+-4.9	1874	1.00 (ref)	1874	1.00 (ref)	1867	1.00 (ref)	1398	1.00 (ref)	1787	1.00 (ref)
5.0-9.9	523	0.97 (0.88-1.07)	523	0.97 (0.87-1.07)	523	0.97 (0.88-1.07)	306	0.94 (0.83-1.06)	496	0.96 (0.87-1.06)
10.0-14.9	220	0.96 (0.83-1.11)	220	0.96 (0.83-1.10)	220	0.96 (0.83-1.11)	133	1.05 (0.87-1.26)	207	0.95 (0.82-1.10)
15.0-19.9	81	0.83 (0.66-1.04)	81	0.83 (0.66-1.04)	81	0.83 (0.66-1.04)	38	0.73 (0.53-1.01)	77	0.83 (0.66-1.05)
≥20.0	76	0.86 (0.68-1.09)	76	0.86 (0.68-1.09)	76	0.87 (0.68-1.10)	34	0.76 (0.54-1.08)	75	0.93 (0.73-1.18)
Trend per 5 kg gain (d)	2327	0.97 (0.93-1.00)	2327	0.96 (0.92-1.00)	2323	0.97 (0.93-1.00)	1579	0.96 (0.91-1.01)	2223	0.97 (0.93-1.01)
Weight change from 25-34 to 45-54 years										
Lost ≥5.0	33	1.31 (0.91-1.90)	33	1.32 (0.91-1.91)	33	1.31 (0.91-1.90)	29	1.43 (0.96-2.11)	32	1.23 (0.86-1.78)
+-4.9	417	1.00 (ref)	417	1.00 (ref)	417	1.00 (ref)	374	1.00 (ref)	389	1.00 (ref)
5.0-9.9	333	1.11 (0.96-1.29)	333	1.12 (0.97-1.29)	333	1.11 (0.96-1.29)	292	1.11 (0.95-1.29)	310	1.11 (0.95-1.28)
10.0-14.9	172	0.95 (0.79-1.14)	172	0.96 (0.80-1.14)	172	0.95 (0.79-1.14)	142	0.91 (0.75-1.10)	155	0.90 (0.74-1.08)
15.0-19.9	87	0.90 (0.71-1.14)	87	0.91 (0.72-1.15)	87	0.90 (0.71-1.14)	76	0.92 (0.72-1.18)	82	0.88 (0.69-1.12)
≥20.0	105	0.97 (0.78-1.22)	105	0.99 (0.79-1.23)	105	0.97 (0.78-1.22)	86	0.93 (0.73-1.19)	99	0.96 (0.76-1.20)
Trend per 5 kg gain (d)	1042	0.97 (0.93-1.01)	1042	0.97 (0.93-1.01)	1042	0.97 (0.93-1.01)	909	0.96 (0.92-1.01)	966	0.97 (0.93-1.01)
Weight change from 35-44 to 45-54 years										
Lost ≥5.0	164	1.11 (0.94-1.31)	164	1.12 (0.95-1.32)	154	1.08 (0.91-1.29)	121	1.03 (0.85-1.25)	132	0.99 (0.83-1.18)
+-4.9	2540	1.00 (ref)	2540	1.00 (ref)	2446	1.00 (ref)	2229	1.00 (ref)	1807	1.00 (ref)
5.0-9.9	858	0.97 (0.90-1.05)	858	0.99 (0.91-1.07)	825	0.97 (0.90-1.05)	710	0.94 (0.86-1.02)	675	0.93 (0.85-1.02)
10.0-14.9	388	1.05 (0.94-1.17)	388	1.08 (0.97-1.21)	356	1.02 (0.91-1.14)	321	1.03 (0.92-1.17)	322	1.03 (0.91-1.16)
15.0-19.9	166	1.08 (0.92-1.26)	166	1.11 (0.95-1.31)	155	1.08 (0.92-1.28)	142	1.10 (0.92-1.31)	137	1.06 (0.89-1.27)
≥20.0	135	0.92 (0.77-1.10)	135	0.96 (0.80-1.15)	116	0.90 (0.74-1.09)	106	0.88 (0.72-1.08)	96	0.89 (0.72-1.09)
Trend per 5 kg gain (d)	3555	0.99 (0.96-1.02)	3555	1.00 (0.97-1.03)	3388	0.98 (0.95-1.01)	3047	0.98 (0.95-1.02)	2670	0.98 (0.95-1.01)

Abbreviations: CI, confidence interval

(a) HRs adjusted for attained age and study cohort, year of birth, age at menarche, age at first birth, number of births, time since last birth, family history of breast cancer, adult height and weight at the start of each weight change age group
(b) HRs additionally adjusted for the number of years between weight assessments
(c) HRs adjusted weight at ages 18-24 years rather than for the weight at the start of each weight change age group
(d) (d) Linear trend per 5kg weight gain fitted across weight change values from ≥0.0 kg

Table S9: Risk of premenopausal breast cancer in relation to weight change between various ages, main results and results restricted to the 5 cohorts that contributed to analyses of weight change at all of the six age groups investigated

			Weight gain category, kg						
			Loss ≥5.0kg	± 4.9kg	Gain 5.0-9.9kg	Gain 10.0-14.9kg	Gain 15.0-19.9 kg	Gain ≥20.0kg	Trend per 5kg weight gain (b)
Ages at weight change	Group (c)	No. of cases	HR (95% CI)(a)	HR (a)	HR (95% CI)(a)	HR (95% CI)(a)	HR (95% CI)(a)	HR (95% CI)(a)	HR (95% CI)(a)
Age 18-24 to 25-34	All	3585	1.09 90.93-1.27)	1.00	0.96 (0.88-1.04)	0.97 (0.87-1.08)	0.93 (0.80-1.08)	0.88 (0.77-1.02)	0.98 (0.96-1.01)
	Subset	3067	1.10 (0.93-1.31)	1.00	0.97 (0.88-1.06)	0.97 (0.86-1.09)	0.99 (0.85-1.16)	0.90 (0.78-1.05)	0.99 (0.96-1.01)
Age 18-24 to 35-44	All	8038	0.92 (0.81-1.04)	1.00	0.98 (0.93-1.04)	0.93 (0.87-0.99)	0.89 (0.82-0.97)	0.85 (0.79-0.92)	0.97 (0.96-0.98)
	Subset	5901	0.93 (0.80-1.07)	1.00	0.97 (0.91-1.04)	0.93 (0.86-1.00)	0.89 (0.81-0.98)	0.85 (0.77-0.92)	0.97 (0.96-0.99)
Age 18-24 to 45-54	All	4073	0.95 (0.78-1.15)	1.00	0.97 (0.89-1.06)	0.96 (0.87-1.06)	0.86 (0.77-0.96)	0.80 (0.73-0.88)	0.96 (0.95-0.98)
	Subset	3213	1.00 (0.80-1.25)	1.00	1.00 (0.90-1.11)	0.98 (0.88-1.09)	0.86 (0.75-0.97)	0.82 (0.73-0.91)	0.97 (0.95-0.98)
Age 25-34 to 35-44	All	2893	1.00 (0.83-1.21)	1.00	0.97 (0.88-1.07)	0.96 (0.83-1.11)	0.83 (0.66-1.04)	0.86 (0.68-1.09)	0.97 (0.93-1.00)
	Subset	2699	1.06 (0.87-1.28)	1.00	0.95 (0.85-1.05)	0.93 (0.80-1.08)	0.84 (0.67-1.07)	0.89 (0.70-1.13)	0.97 (0.93-1.01)
Age 25-34 to 45-54	All	1147	1.31 (0.91-1.90)	1.00	1.11 (0.96-1.29)	0.95 (0.79-1.14)	0.90 (0.71-1.14)	0.97 (0.78-1.22)	0.97 (0.93-1.01)
	Subset	1147	1.31 (0.91-1.90)	1.00	1.11 (0.96-1.29)	0.95 (0.79-1.14)	0.90 (0.71-1.14)	0.97 (0.78-1.22)	0.97 (0.93-1.01)
Age 35-44 to 45-54	All	4251	1.11 (0.94-1.31)	1.00	0.97 (0.90-1.05)	1.05 (0.94-1.17)	1.08 (0.92-1.26)	0.92 (0.77-1.10)	0.99 (0.96-1.02)
	Subset	2955	1.07 (0.88-1.31)	1.00	0.93 (0.82-1.02)	1.03 (0.91-1.16)	1.11 (0.93-1.32)	0.93 (0.76-1.15)	0.99 (0.95-1.02)

Abbreviations: CI, confidence interval; ER, oestrogen-receptor; HR, hazard ratio; PR, progesterone-receptor

(a) HRs are adjusted for attained age, cohort, year of birth, adult height, weight at start of age range, age at menarche, age at first birth, number of births, time since last birth and family history of breast cancer

(b) Including subjects with weight gain only

(c) All cohorts vs. the 5 cohorts that contributed to analyses of weight change at all of the six age groups investigated (NHS, NHSII, USRTC, SWLHS, BWHS)

Figure S1: Trajectory modelling using 3 time points and 6 trajectories across age range 18-54 years using 15 cohorts. Solid lines represent the estimated trajectories, dots represent observed trajectory mean value at specific age and legend show the estimated percentages of subjects contributing to that trajectory. Model includes quadratic terms.

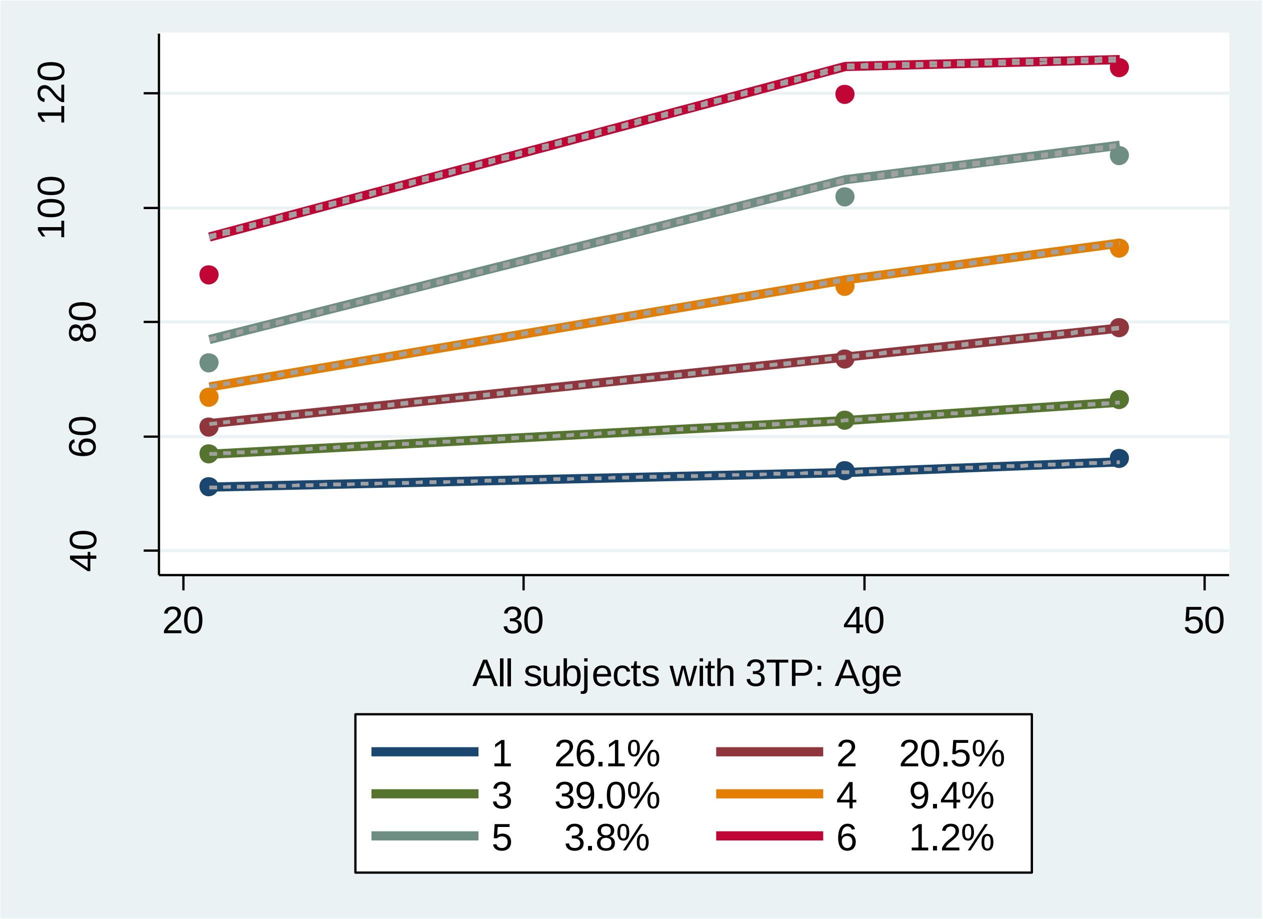

Descriptives by trajectory

Trajectory group	N subjects	1st weight assessment		2nd weight assessment		3rd weight assessment	
		Median weight	Median age	Median weight	Median age	Median weight	Median age
1	102,885	50.8	18.0	53.6	41.2	55.8	49.0
2	81,775	61.2	18.0	73.0	40.0	79.5	48.0
3	163,119	56.8	18.0	62.7	40.0	65.9	48.2
4	37,506	65.8	18.0	86.4	39.0	93.1	47.6
5	15,372	72.6	18.0	102.3	38.3	109.1	47.0
6	4,945	86.3	18.0	120.3	36.5	125.0	45.0
Totals	405,602	56.8	18.0	63.6	40.0	68.0	48.1

Traj = trajectory group, N= number, 1st = 1st weight assessment etc., median weight is in kilograms, median age is years

Figure S2: Trajectory modelling using 8 time points and 6 trajectories across age range 18-54 years using 3 cohorts. Solid lines represent the estimated trajectories, dots represent observed trajectory mean value at specific age and legend show the estimated percentages of subjects contributing to that trajectory. Model includes quadratic and cubic terms.

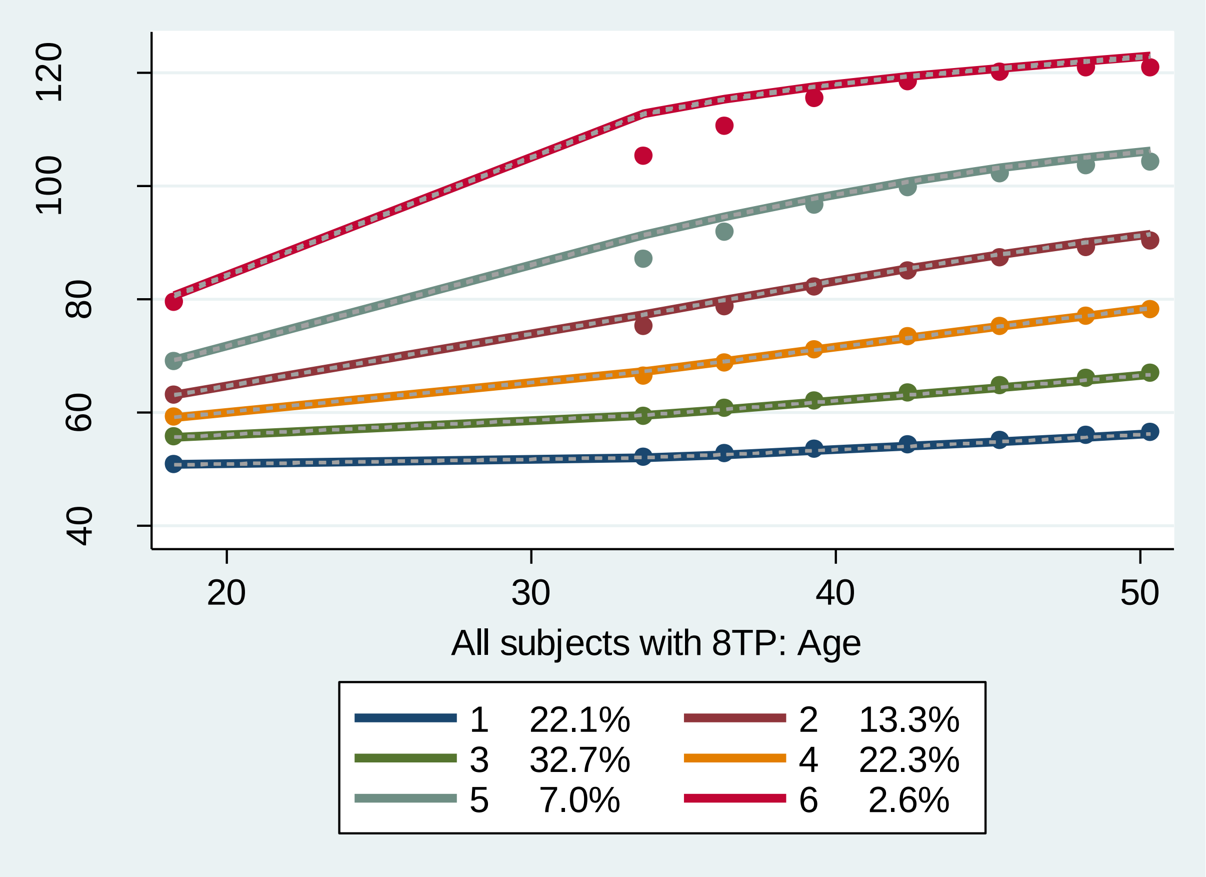

Descriptives by trajectory

Traj	N subjects	1st		2nd		3rd		4th		5th		6th		7th		8th	
		kg	age	kg	age	kg	age	kg	age	kg	age	kg	age	kg	age	kg	age
1	27,001	50.0	18.0	52.3	34.7	52.7	37.5	53.6	40.7	54.5	43.8	55.5	47.0	56.4	50.0	56.8	52.2
2	16,341	61.4	18.0	75.0	33.0	78.6	35.8	81.8	38.8	85.0	42.0	86.7	45.0	89.1	47.7	90.8	49.8
3	40,375	54.5	18.0	59.1	34.2	60.9	37.0	61.8	40.1	63.6	43.3	64.5	46.5	65.9	49.5	66.8	51.6
4	27,329	58.2	18.0	65.9	33.5	68.2	36.3	70.5	39.4	72.7	42.8	75.0	45.8	77.2	48.5	77.7	50.7
5	8,629	68.1	18.0	86.4	32.2	90.9	35.0	96.4	38.0	100.0	41.2	102.3	44.2	104.4	47.0	104.5	49.0
6	3,171	77.3	18.0	104.5	31.0	111.2	33.8	114.4	36.9	118.2	39.9	120.3	43.0	121.2	45.9	121.8	48.0
Total	122,846	56.8	18.0	61.4	33.8	63.6	36.7	65.0	39.8	66.8	43.0	68.2	46.0	69.9	48.9	70.5	51.0

Traj = trajectory group, N= number, 1st = 1st weight assessment etc., kg= median weight (kg), age= median age

Figure S3: Relative risk of premenopausal breast cancer in relation to weight change between various ages, by 2.5kg increments

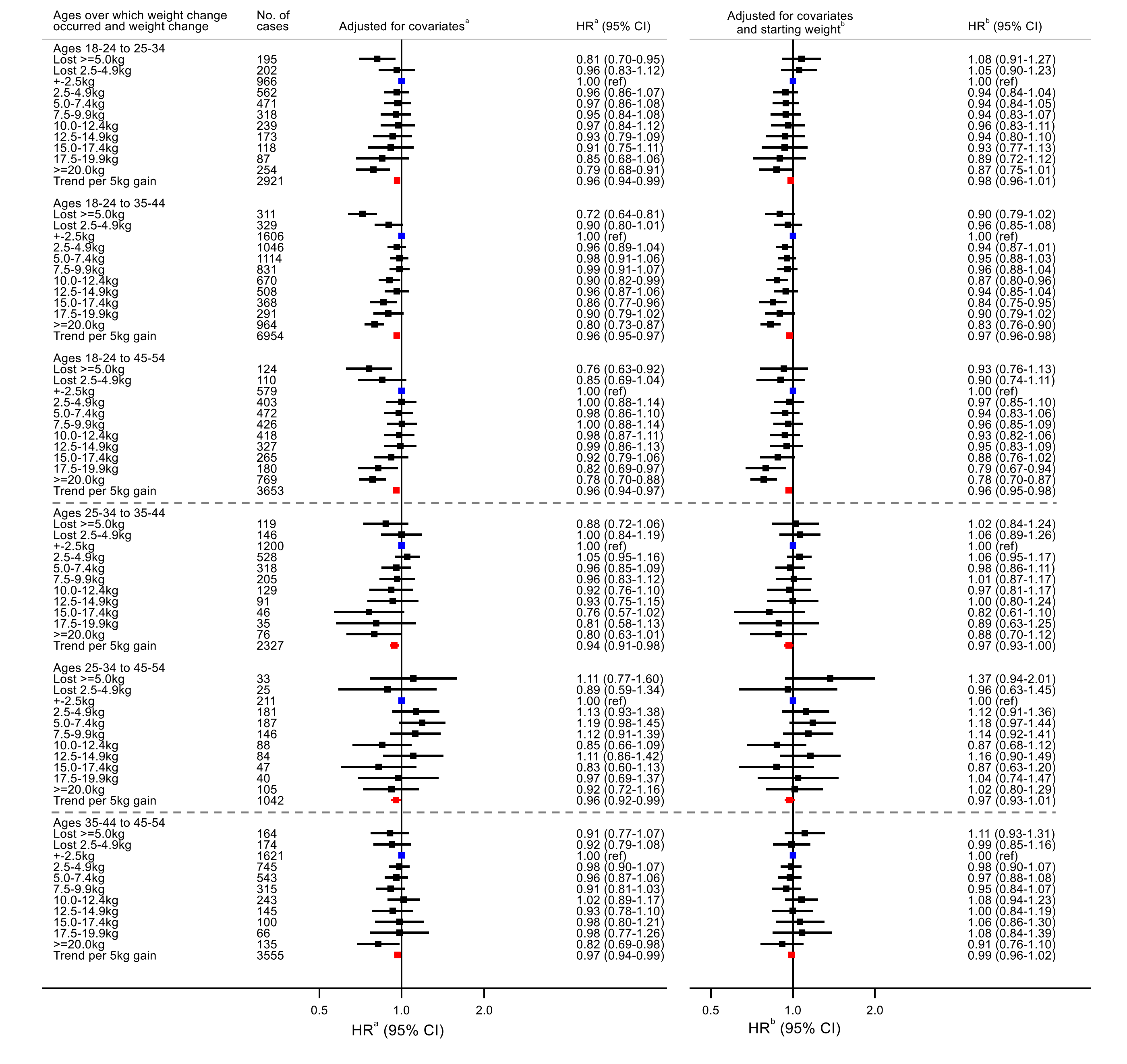

Abbreviations: HR, hazard ratio; CI, confidence interval

(a) Adjusted for attained age, cohort, year of birth, adult height, age at menarche, age at first birth, number of births, time since last birth and family history of breast cancer

(b) Adjusted for covariates in (a) plus weight at start of age range

Figure S4: Risk of premenopausal breast cancer in relation to weight gain per 5kg between various ages, by breast cancer intrinsic subtype

Weight change category by intrinsic subtype	No. of cases	HR[a] per 5kg weight gain (95% CI)
**Age 18-24 to 25-34**		
Luminal A-like, all	659	0.94 (0.90-0.99)
Luminal B-like, all	347	1.01 (0.95-1.09)
---Luminal B HER2-	88	1.02 (0.87-1.20)
---Luminal B HER2+	143	1.00 (0.89-1.12)
Non-luminal, all	419	1.06 (1.00-1.12)
---HER2-enriched	65	0.95 (0.80-1.13)
---Triple-negative	149	1.05 (0.95-1.17)
**Age 18-24 to 35-44**		
Luminal A-like, all	1394	0.94 (0.91-0.97)
Luminal B-like, all	822	0.94 (0.90-0.98)
---Luminal B HER2-	245	0.94 (0.87-1.01)
---Luminal B HER2+	312	0.96 (0.90-1.02)
Non-luminal, all	881	1.03 (1.00-1.07)
---HER2-enriched	143	1.00 (0.93-1.08)
---Triple-negative	330	0.98 (0.93-1.04)
**Age 18-24 to 45-54**		
Luminal A-like, all	913	0.97 (0.94-1.00)
Luminal B-like, all	483	0.94 (0.90-0.98)
---Luminal B HER2-	151	0.93 (0.86-0.99)
---Luminal B HER2+	188	0.97 (0.91-1.04)
Non-luminal, all	448	1.00 (0.96-1.04)
---HER2-enriched	73	1.07 (0.98-1.16)
---Triple-negative	185	1.00 (0.94-1.07)
**Age 25-34 to 35-44**		
Luminal A-like, all	587	0.94 (0.87-1.01)
Luminal B-like, all	292	0.96 (0.86-1.07)
---Luminal B HER2-	81	0.77 (0.61-0.98)
---Luminal B HER2+	120	1.06 (0.92-1.22)
Non-luminal, all	309	1.08 (1.00-1.16)
---HER2-enriched	56	1.16 (1.00-1.35)
---Triple-negative	124	0.97 (0.86-1.09)
**Age 25-34 to 45-54**		
Luminal A-like, all	361	0.93 (0.87-1.00)
Luminal B-like, all	147	0.91 (0.81-1.01)
---Luminal B HER2-	38	0.85 (0.69-1.06)
---Luminal B HER2+	58	0.96 (0.81-1.13)
Non-luminal, all	123	0.97 (0.87-1.08)
---HER2-enriched	28	1.15 (0.94-1.40)
---Triple-negative	51	0.98 (0.85-1.12)
**Age 35-44 to 45-54**		
Luminal A-like, all	916	1.02 (0.97-1.07)
Luminal B-like, all	505	0.97 (0.90-1.04)
---Luminal B HER2-	133	0.96 (0.84-1.10)
---Luminal B HER2+	178	1.07 (0.97-1.18)
Non-luminal, all	454	0.95 (0.87-1.03)
---HER2-enriched	74	1.08 (0.93-1.25)
---Triple-negative	181	0.99 (0.87-1.12)

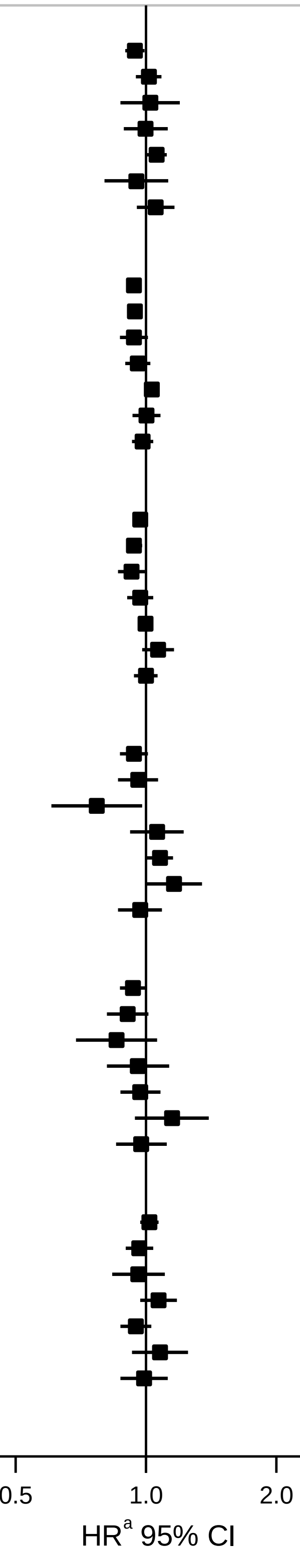

Abbreviations: CI, confidence interval; HR, hazard ratio; HER2, human epidermal growth factor receptor-2

Luminal A-like: ER+PR+HER2- ; Luminal B-like, all: All ER+ and/or PR+ tumours that are not ER+PR+HER2- with subtypes Luminal B-like, HER2-: ER+PR−HER2- and ER−PR+HER2- and Luminal B-like, HER2+: [ER+ and/or PR+] and HER2+ ; Non-luminal, all: ER−PR− regardless of HER2 status with subtypes HER2-enriched: ER−PR−HER2+ and Triple-negative: ER−PR−HER2-

(a) HRs represent linear trend per 5 kg weight gain and are adjusted for attained age, cohort, year of birth, adult height, weight at start of age range, age at menarche, age at first birth, number of births, time since last birth and family history of breast cancer. Estimates were obtained from two Augmentation models[8], one including Luminal A-like, Luminal B-like and non-luminal breast cancer as endpoints with tests for heterogeneity in effect by tumour type: for weight change 18-24 to 25-34 , p=0.54; 18-24 to 35-44, p=0.09; 18-24 to 45-54, p=0.77; 25-34 to 35-44, p=0.26; 25-34 to 45-54, p<0.001, 35-44 to 45-54, p=0.40 Estimates for subtypes of luminal B-like and non-luminal breast cancer were obtained from a second model fitting Luminal A-like, luminal B-like HER2+, luminal B-like HER2-, HER2-enriched and triple-negative breast cancer as endpoints.

Figure S5: Risk of premenopausal breast cancer in relation to the annual rate of weight change between various age groups

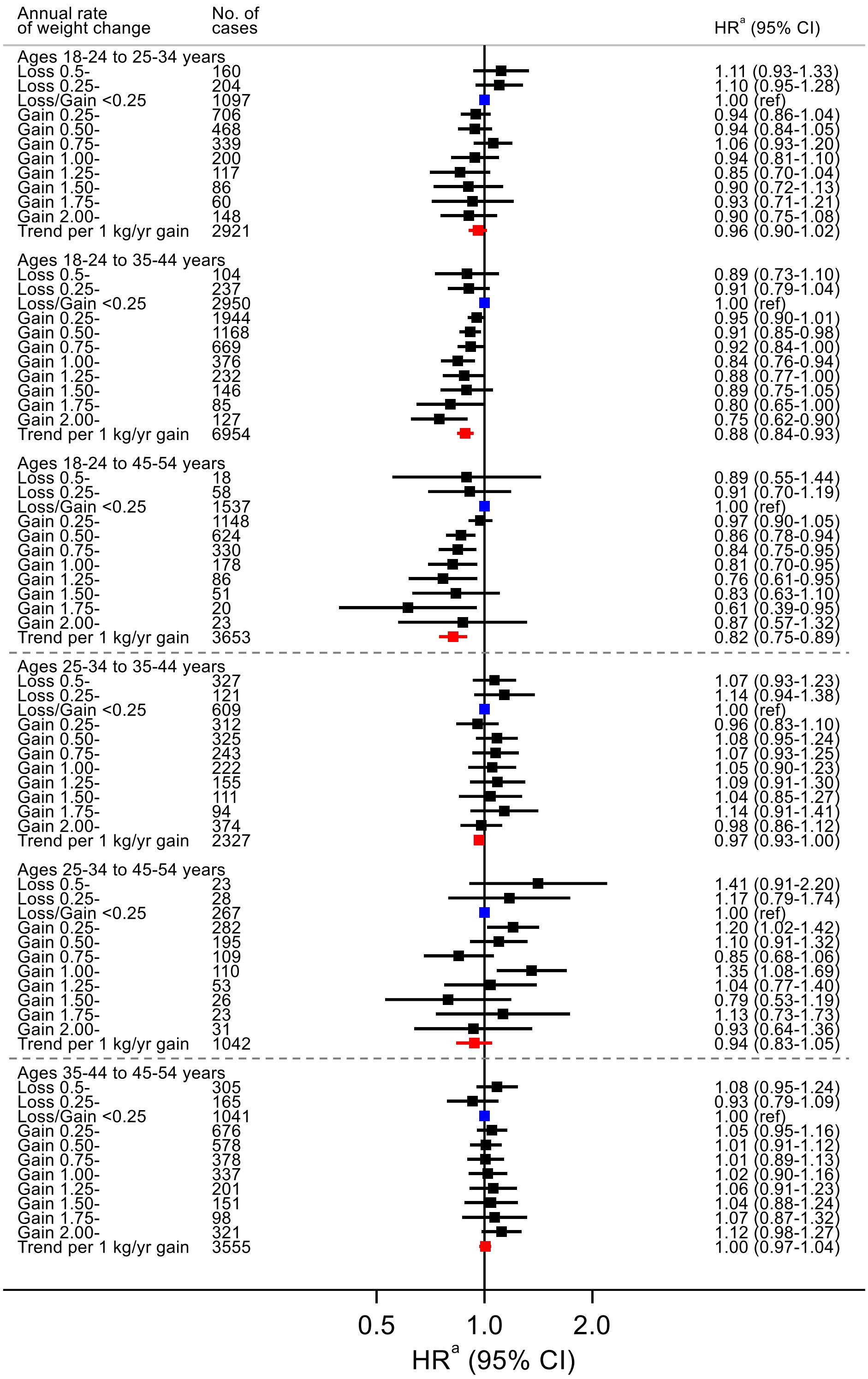

Abbreviations: HR, hazard ratio; CI, confidence interval

(a) HRs are adjusted for attained age, cohort, year of birth, adult height, weight at start of age range, age at menarche, age at first birth, number of births, time since last birth and family history of breast cancer. Hazard ratios were very similar when additionally adjusting for the time intervals between the weight assessments.

References

1. Nagin DJ, BL; Passos, LV, Tremblay, RE;. Group-based multi-trajectory modeling. *Statistics in Medical Research* 2016.
2. StataCorp, ed. *Stata Statistical Software: Release 14*ed. College Station, Texas: StataCorp LP, 2015.
3. Goldhirsch A, Winer EP, Coates AS, Gelber RD, Piccart-Gebhart M, Thurlimann B, Senn HJ, Panel m. Personalizing the treatment of women with early breast cancer: highlights of the St Gallen International Expert Consensus on the Primary Therapy of Early Breast Cancer 2013. *Ann Oncol* 2013;24:2206-23.
4. Cox DR. Regression Models and Life-Tables. *Journal of the Royal Statistical Society* 1972;34:187-220.
5. Barlow WE, Ichikawa L, Rosner D, Izumi S. Analysis of case-cohort designs. *J Clin Epidemiol* 1999;52:1165-72.
6. Smith-Warner SA, Spiegelman D, Ritz J, Albanes D, Beeson WL, Bernstein L, Berrino F, van den Brandt PA, Buring JE, Cho E, Colditz GA, Folsom AR, Freudenheim JL, Giovannucci E, Goldbohm RA, Graham S, Harnack L, Horn-Ross PL, Krogh V, Leitzmann MF, McCullough ML, Miller AB, Rodriguez C, Rohan TE, Schatzkin A, Shore R, Virtanen M, Willett WC, Wolk A, Zeleniuch-Jacquotte A, Zhang SM, Hunter DJ. Methods for pooling results of epidemiologic studies: the Pooling Project of Prospective Studies of Diet and Cancer. *AmJEpidemiol* 2006;163:1053-64.
7. Buse A. The Likelihood Ratio, Wald, and Lagrange Multiplier Tests - an Expository Note. *Am Stat* 1982;36:4.
8. Lunn M, McNeil D. Applying Cox regression to competing risks. *Biometrics* 1995;51:524-32.
9. Nichols HB, Schoemaker MJ, Wright LB, McGowan C, Brook MN, McClain KM, Jones ME, Adami HO, Agnoli C, Baglietto L, Bernstein L, Bertrand KA, Blot WJ, Boutron-Ruault MC, Butler L, Chen Y, Doody MM, Dossus L, Eliassen AH, Giles GG, Gram IT, Hankinson SE, Hoffman-Bolton J, Kaaks R, Key TJ, Kirsh VA, Kitahara CM, Koh WP, Larsson SC, Lund E, Ma H, Merritt MA, Milne RL, Navarro C, Overvad K, Ozasa K, Palmer JR, Peeters PH, Riboli E, Rohan TE, Sadakane A, Sund M, Tamimi RM, Trichopoulou A, Vatten L, Visvanathan K, Weiderpass E, Willett WC, Wolk A, Zeleniuch-Jacquotte A, Zheng W, Sandler DP, Swerdlow AJ. The Premenopausal Breast Cancer Collaboration: A Pooling Project of Studies Participating in the National Cancer Institute Cohort Consortium. *Cancer Epidemiol Biomarkers Prev* 2017;26:1360-9.
